# Amlexanox Ameliorates Traumatic Brain Injury by Restoring Autophagy-Lysosomal Function via cAMP Signaling Modulation

**DOI:** 10.7150/ijbs.111216

**Published:** 2025-07-06

**Authors:** Seo Young Woo, Min Kyu Park, A Ra Kho, Hyun Wook Yang, Hyun Ho Jung, Jaewoo Shin, Minwoo Lee, Ha Na Kim, Jae Young Koh, Bo Young Choi, Sang Won Suh

**Affiliations:** 1 Department of Physiology, Hallym University College of Medicine, Chuncheon 24252, Republic of Korea.; 2 Neuroregeneration and Stem Cell Programs, Institute for Cell Engineering, Johns Hopkins University School of Medicine, Baltimore, MD 21205, USA.; 3 Department of Neurology, Johns Hopkins University School of Medicine, Baltimore, MD 21205, USA.; 4 Medical Device Development Center, Daegu-Gyeongbuk Medical Innovation Foundation (K-MEDI Hub), Daegu 41061, Republic of Korea.; 5 Department of Neurology, Hallym Neurological Institute, Hallym University Sacred Heart Hospital, Anyang 14068, Republic of Korea.; 6 Neural Injury Lab, Biomedical Research Center, Asan Institute for Life Sciences, Asan Medical Center, Seoul, Korea.; 7 Department of Physical Education, Hallym University, Chuncheon 24252, Republic of Korea.; 8 Institute of Sport Science, Hallym University, Chuncheon 24252, Republic of Korea.

**Keywords:** traumatic brain injury (TBI), amlexanox (AMX), phosphodiesterase (PDE), protein kinase A (PKA), cyclic adenosine monophosphate (cAMP), lysosomal dysfunction, autophagy, neuronal death

## Abstract

Traumatic brain injury (TBI) disrupts cellular homeostasis through lysosomal dysfunction, oxidative stress, and impaired autophagy, contributing to neuronal degeneration. Despite advances in our understanding of these mechanisms, effective therapeutic options remain limited. This study investigates amlexanox (AMX), a broad-spectrum phosphodiesterase (PDE) inhibitor, as a potential treatment for TBI-induced neuronal damage. AMX not only increases cyclic adenosine monophosphate (cAMP) levels by inhibiting multiple PDE isoforms but also exhibits anti-inflammatory properties by suppressing pro-inflammatory cytokine production and glial activation via NF-κB and STAT3 pathway inhibition. This dual pharmacological profile suggests a multifaceted therapeutic potential for brain injury. High-throughput screening of an FDA-approved drug library identified AMX as an agent that restores lysosomal acidity through protein kinase A (PKA) activation in primary neuron cultures. *In vitro* scratch assays demonstrated that AMX enhances lysosomal function, reduces dendritic loss, and promotes neuronal survival. Using a controlled cortical impact model, *in vivo* experiments revealed that AMX alleviates oxidative and endoplasmic reticulum stress, suppresses neuroinflammation by reducing microglial and astrocytic activation, and preserves neuronal viability in the hippocampus. Behavioral assessments confirmed significant improvements in cognitive and neurological deficits following TBI. These findings establish that AMX is a promising therapeutic agent that restores lysosomal function and mitigates TBI-induced neuronal damage through multi-target PDE inhibition and anti-inflammatory actions.

## Introduction

Traumatic brain injury (TBI) is a major global health issue and a leading cause of long-term disability, often resulting from external forces such as accidents, falls, and sports injuries 1, 2. These events directly damage the brain, which can lead to temporary or permanent functional impairments and, in severe cases, death 2, 3. TBI consists of two phases: the primary injury, which involves immediate physical damage, and the secondary injury, characterized by a series of complex cellular and molecular processes that exacerbate the initial damage 2. These secondary processes include neuronal loss, inflammation, oxidative stress, and disruptions in the blood-brain barrier (BBB), which collectively contribute to progressive neurodegeneration and cognitive decline 4-6.

One key contributor to secondary injury is oxidative stress, a condition arising from the overproduction of reactive oxygen species (ROS) 5, 7, 8. Excess ROS levels damage cellular components, disrupt signaling pathways, and intensify inflammatory responses 7, 8. Another significant factor is endoplasmic reticulum (ER) stress, which occurs when the ER's ability to fold and process proteins becomes overwhelmed 6. This leads to an accumulation of misfolded proteins, activating a cellular response aimed at restoring balance 6. However, prolonged ER stress can trigger cell death, further worsening TBI outcomes 9, 10. The interplay between oxidative stress and ER stress creates a toxic environment that exacerbates neuronal damage and impairs recovery 9, 11.

Zinc dysregulation is another critical aspect of TBI pathology. Zinc is essential for various brain functions, including neurotransmission and enzymatic activity 12, 13. However, TBI can disturb zinc homeostasis, resulting in its excessive release and accumulation in neurons. This imbalance disrupts lysosomal and autophagic processes, crucial for clearing damaged cellular components 14, 15. Dysfunctional lysosomes and impaired autophagy contribute to oxidative stress and the activation of harmful pathways, worsening neuronal damage 16.

Targeting these interconnected mechanisms, such as oxidative stress, ER stress, and zinc dysregulation, is a promising approach for mitigating the secondary injury effects of TBI 17-19. The lysosome-autophagy pathway, a critical system for cellular maintenance, is key in this context and represents a potential therapeutic target 12.

Phosphodiesterases (PDEs) are a family of enzymes involved in regulating cellular signaling pathways, including those affected by TBI 20, 21. By degrading cyclic nucleotides such as cyclic adenosine monophosphate (cAMP), PDEs influence varied cellular functions 21. Among these, PDE4 is particularly significant in the brain due to its role in modulating inflammatory and neuroprotective pathways 20-22. Dysregulated PDE activity during TBI reduces cAMP levels, impairing the protective mechanisms that combat inflammation and oxidative stress 23-25. While selective PDE4 inhibitors have shown therapeutic promise, the broader inhibition of multiple PDE isoforms could address more varied pathological processes, offering a more comprehensive treatment strategy 25, 26.

Amlexanox (AMX), a drug traditionally used to treat inflammatory conditions, has recently been identified as a broad-spectrum PDE inhibitor, specifically a PDE4 inhibitor 27. By increasing cAMP levels, AMX activates protective pathways that mitigate inflammation and oxidative stress 28, 29. Preclinical studies have suggested that AMX may also enhance lysosomal function, reduce neuroinflammation, and improve outcomes in conditions involving complex cellular damage 28, 30.

Despite its therapeutic potential, the effects of AMX on TBI-related lysosomal dysfunction, zinc dysregulation, and neuronal damage remain underexplored 31. In this study, we investigate the ability of AMX to simultaneously target multiple interconnected pathological pathways in TBI 28. By employing both cellular and animal models, we study the molecular mechanisms underlying AMX's protective effects and assess its efficacy in improving cognitive and neurological outcomes following TBI 28. We also hypothesize that AMX, a broad-spectrum PDE inhibitor, exerts neuroprotective effects against TBI-induced neuronal damage by modulating inflammatory and oxidative stress pathways.

## Material and Methods

### Ethics committee approval and animal care

This study used male Sprague-Dawley rats weighing 300-350 g and aged 8 weeks, obtained from DBL Co. (Chungcheongbuk-do, Republic of Korea). The rats were housed in a controlled environment with a temperature of 20 ± 2 °C, a relative humidity of 55 ± 5%, and a 12-hour light/dark cycle. They were provided ad libitum access to food and water. To minimize stress associated with transportation, animals underwent a one-week acclimatization period before the start of the experiments. All experimental procedures were conducted in compliance with the guidelines of the National Institutes of Health for the care and use of laboratory animals and were approved by the Hallym University Institutional Animal Care and Use Committee (protocol # Hallym 2022-23, approved on July 15, 2022). This manuscript follows the principles outlined in the Animal research: reporting *in vivo* experiments (ARRIVE) guidelines.

### Primary neuron culture

Primary hippocampal neurons were obtained from embryonic day 18 (E18) Sprague-Dawley rat embryos. Pregnant rats were anesthetized using 2-3% isoflurane in a gas mixture of 70% nitrous oxide and 30% oxygen. Following euthanasia, the hippocampi were carefully dissected, minced, and enzymatically dissociated using TrypLE Express (Gibco, NY, USA) for 10 minutes at 37°C. The tissue was further mechanically dissociated using fire-polished glass pipettes, and the resulting cell suspension was filtered through a 70 μm cell strainer to remove debris. Neurons were seeded at a density of 3 × 10^5 cells per well in 24-well plates pre-coated with poly-L-lysine (Sigma-Aldrich, Saint Louis, MO, USA). Cells were maintained in neurobasal medium supplemented with B-27 (Thermo Fisher Scientific, Waltham, MA, USA), GlutaMAX (Gibco), and penicillin-streptomycin. The cultures were incubated at 37.5°C in a humidified atmosphere containing 5% CO₂. After seven days of growth, neurons were fixed with 4% paraformaldehyde (PFA) for 15 minutes at room temperature and subsequently stored at 4°C until their further use for immunostaining.

### HTS of FDA-approved drug library for PDE inhibition

To evaluate the PDE inhibitory activity of AMX and other compounds, the SCREEN-WELL® FDA v. 2.0 Approved Drug Library (Enzo Biochem, Inc., NY, USA) was utilized in a 96-well plate format. PDE isoform activity was assessed using the PDELight™ HTS cAMP Phosphodiesterase Assay (LONZA, MD, USA), which measures luminescence as a marker of cAMP degradation. Each well contained a 50 μL reaction mixture, which included 5 μM of cAMP as the substrate, the specific PDE enzyme, and the test compound at varying concentrations. The reactions were incubated at 30°C for 60 minutes. Luminescence was then measured using a microplate reader to quantify PDE activity, with reduced luminescence indicating greater PDE inhibition.

### PKA colorimetric activity assay

PKA activity was assessed using a microplate-based kinase activity assay. Each well contained reaction buffer, a purified PKA enzyme, and test samples, followed by adding 10 μL of adenosine triphosphate (ATP) solution to initiate the reaction. Plates were incubated at 30°C for 90 minutes, before being washed four times with wash buffer to remove unbound components. Next, 25 μL each of phospho-PKA substrate antibody and horseradish peroxidase (HRP)-conjugated secondary antibody (donkey anti-rabbit IgG) were added to each well. The plates were incubated at room temperature (RT) for 60 minutes under continuous shaking to ensure uniform binding. To detect the phosphorylated substrate, 100 μL of tetramethylbenzidine (TMB) substrate was added to each well, followed by a 30-minute incubation at RT. The reaction was terminated by adding stop solution, and the absorbance was measured at 450 nm by using a microplate reader to quantify PKA activity.

### Lysosomal pH detection

Primary neurons were cultured on glass slides pre-coated with poly-L-lysine. To assess lysosomal acidity, cells were stained with Lysosensor DND-189, a pH-sensitive fluorescent dye (Invitrogen, MA, USA). Staining was performed by incubating the neurons with the dye in their growth medium for 30 minutes in a humidified CO₂ incubator. After staining, cells were transferred to Live Cell Imaging Solution (Thermo Fisher Scientific) to maintain viability during imaging. Fluorescence was captured using a LSM710 confocal microscope (Carl Zeiss, Oberkochen, Germany), with excitation and emission wavelengths set at 440 nm and 520 nm, respectively. The intensity of lysosomal fluorescence, indicative of pH levels, was quantified using specialized imaging software.

### *In vitro* TBI model (scratch assay)

A scratch injury model was used to mimic TBI *in vitro*. Primary neurons were cultured for 7 days, after which a 5 × 5 scratch pattern with 3 mm intervals was created on the monolayer using a 100 μL pipette tip. To remove cellular debris, the culture medium was carefully aspirated and replaced with fresh neurobasal medium. Neurons in the experimental group were treated with neurobasal medium supplemented with 10 μM of AMX for 24 hours, while the control group received neurobasal medium without the drug. After the treatment period, neuronal damage and recovery were assessed using appropriate evaluation methods.

### Cell viability analysis

Following scratch injury, neuronal viability was assessed using the EZ-Cytox cell viability assay kit (DoGenBio Co., Seoul, Korea). A mixture of 100 μL of culture medium and EZ-Cytox reagent was added to each well. The plates were incubated at 37°C for 2 hours to allow the reagent to interact with viable cells. After incubation, absorbance was measured at 450 nm using a microplate reader (Spectramax, Molecular Devices Co., CA, USA). Viability data were normalized to the untreated control group to determine relative cell viability.

### Western blot analysis (*in vitro*)

Twenty-four hours after the scratch injury, cells were lysed using RIPA buffer (IBS-BR002, iNtRON, Republic of Korea) supplemented with protease and phosphatase inhibitors (Sigma, USA). The lysed cells were scraped, briefly vortexed for 10 seconds, and kept on ice for three 10-minute intervals to ensure complete lysis. The samples were then centrifuged at 14,000 rpm for 30 minutes at 4°C, and the supernatant was collected for protein concentration measurement using the Bradford assay. Proteins (20 μg per sample) were prepared through mixing with SDS loading buffer and separated via SDS-PAGE. After electrophoresis, proteins were transferred onto a PVDF membrane. To block nonspecific binding, the membrane was incubated in 5% skim milk or 3% BSA for 2 hours at room temperature. Primary antibodies, including PDE4 (FabGennix, 1:500), LAMP2 (Sigma, 1:1000), and β-actin (Cell Signaling, 1:10,000), were applied to the membrane and incubated overnight at 4°C. The membrane was then washed three times with TBS-T (Bio-Rad, Hercules, CA, USA) to remove unbound antibodies and subsequently incubated with HRP-conjugated secondary antibodies (anti-mouse IgG or anti-rabbit IgG, Ab Frontier, 1:5000) for 1 hour at room temperature on a shaker. Protein detection was performed using a chemiluminescence bioimaging device (Amersham Imager 680, Cytiva, MA, USA), and the results were analyzed to determine protein expression levels.

### cAMP analysis

Six hours after the scratch assay, the culture media were replaced with 0.1 M HCl to stop cellular activity. The cells were incubated at room temperature for 20 minutes to allow the acid extraction of cAMP. Adherent cells were then detached using a cell scraper and further dispersed via gentle pipetting to ensure homogeneity. The cell suspension was centrifuged at 1,000 × g for 10 minutes at 4°C, and the supernatant containing cAMP was carefully collected. The quantification of cAMP levels was performed using a cAMP ELISA kit (Cayman Chemical Co., USA), according to the manufacturer's protocol.

### p-PKA analysis

Six hours after the scratch assay, cellular activity was terminated by replacing the culture medium with 0.1 M HCl. Cell lysates were then collected and analyzed for PKA activity using a commercial PKA kinase activity assay kit (Abcam, ab139435), following the manufacturer's protocol. Equal amounts of protein (10-50 μg per well) were loaded onto assay plates pre-coated with a PKA-specific substrate. The kinase reaction was initiated by adding an ATP-containing buffer and incubating the plates at 30°C for 90 minutes. Substrate phosphorylation was detected using a phospho-specific primary antibody and an HRP-conjugated secondary antibody. After chromogenic substrate development, absorbance was measured at 450 nm using a microplate reader (Synergy HTX Multi-Mode Reader, BioTek Instruments, Winooski, VT, USA). PKA activity was quantified as optical density (OD) values and expressed relative to control samples.

### *In vivo* TBI model (controlled cortical impact)

A controlled cortical impact (CCI) model was used to induce TBI in rats. Animals were anesthetized with 1-1.5% isoflurane in a 30:70 oxygen-to-nitrous oxide mixture and securely positioned in a stereotaxic frame. A 3 mm craniotomy was performed using a portable drill at a site 2.8 mm lateral to the midline and 3.0 mm posterior to Bregma. TBI was induced using an Impact One™ Stereotaxic Impactor (Leica Biosystems) equipped with a 3 mm flat-tip, set to deliver an impact at a velocity of 5 m/s and a depth of 3 mm. Throughout the procedure, body temperature was maintained at 36-37.5°C using a homeothermic blanket system to prevent hypothermia. AMX (100 mg/kg; Arctom Scientific) was administered intraperitoneally either 3 or 24 hours after TBI, followed by once-daily injections (100 mg/kg, i.p.) for two weeks. At designated time points (3 hours, 24 hours, or 2 weeks post-injury), animals were euthanized via transcardial perfusion with saline, followed by 4% paraformaldehyde (PFA). Brains were post-fixed in 4% PFA for 1 hour, cryoprotected in 30% sucrose, and sectioned at a 30 μm thickness using a cryostat microtome (CM1850; Leica, Wetzlar, Germany). Brain sections were stored in cryoprotectant solution at 4°C until further analysis.

### Evaluation of neuronal death

Neuronal death was assessed 24 hours after TBI via Fluoro-Jade B (FJB) staining, a method designed for detecting degenerating neurons. Brain sections were mounted on gelatin-coated slides and rehydrated sequentially in 100% ethanol, 70% ethanol, and distilled water. The slides were then incubated in 0.06% potassium permanganate solution for 15 minutes to enhance staining specificity, followed by staining in 0.001% FJB solution (Histo-Chem Inc., Jefferson, AR, USA) for 30 minutes. After staining, sections were rinsed three times in distilled water (1 minute each), cleared in xylene, and coverslipped using DPX mounting medium (Sigma-Aldrich, Munich, Germany). Neuronal degeneration was visualized under a Zeiss LSM 710 confocal microscope (Carl Zeiss, Oberkochen, Germany) with an excitation wavelength of 450-490 nm. FJB-positive cells were quantified in hippocampal subregions, including CA1 (Cornu Ammonis 1), CA3 (Cornu Ammonis 3), GCL (granule cell layer), and the hilus.

### Immunofluorescence staining

Immunofluorescence staining was performed to evaluate the effects of AMX treatment at 3 and 24 hours post-TBI. Brain tissues were initially treated with 1.2% hydrogen peroxide at room temperature for 15 minutes to block endogenous peroxidase activity. Following this step, the tissues were washed three times in PBS for 10 minutes each. The tissues and fixed primary neurons were incubated overnight at 4°C with primary antibodies diluted in antibody diluent. The primary antibodies used included MAP2 (1:500, Millipore), NeuN (1:500, Millipore), PDE4 (1:250, FabGennix), Iba-1 (1:500, Abcam), CD86 (1:100, Bio-Rad), GFAP (1:1000, Abcam), C3 (1:300, Invitrogen), 4HNE (1:500, Alpha Diagnostic Intl.), p-PKA (1:1000, Abcam), and phospho-p38 MAPK (1:400, Cell Signaling). After incubation with primary antibodies, tissues were washed in PBS and incubated for 2 hours at room temperature on a shaker with fluorescent-conjugated secondary antibodies (1:250, Invitrogen). All samples were counterstained with DAPI (1:1000, Invitrogen) to visualize cell nuclei. The stained tissues were mounted on gelatin-coated slides, air-dried, and coverslipped using DPX mounting medium. To evaluate the fluorescent signals of labeled proteins, observations were performed using a Zeiss LSM 710 confocal microscope (Carl Zeiss) with sequential scanning mode for DAPI and Alexa 488 and 594. Stacks of images (1024 × 1024 pixels) from consecutive slices of 0.5-0.8 μm in thickness were obtained by averaging fifteen scans per slice, processed using ZEN 2 (blue edition, Carl Zeiss).

### Immunohistochemistry

Immunohistochemistry was performed to evaluate the effects of AMX treatment at 24 hours and 2 weeks post-TBI. Brain tissues were prepared following a protocol similar to immunofluorescence staining. Sections were incubated overnight at 4°C with NeuN primary antibody (1:500, Millipore) to label neuronal nuclei. The next day, sections were washed and incubated with a secondary IgG antibody (1:250, Vector) for 2 hours at room temperature on a shaker. To assess endogenous IgG leakage, additional sections were treated with the IgG antibody (1:250, Vector) and then incubated in an avidin-biotin-peroxidase complex (ABC) solution for 2 hours at room temperature. Immunoreactivity was visualized by applying a 3,3′-diaminobenzidine (DAB) solution (Sigma-Aldrich), prepared in 0.01 M PBS with 0.015% H₂O₂, for 3 minutes to produce a brown precipitate that indicated antibody binding. Stained sections were mounted on gelatin-coated slides, air-dried, and sealed with Canada balsam (Junsei Chemical, Tokyo, Japan) as the mounting medium. Observations were conducted using a light microscope (Olympus upright microscope IX70, Olympus, Tokyo, Japan).

### Western blot analysis (*in vivo*)

Hippocampal samples were homogenized in a lysis buffer containing RIPA buffer (IBS-BR002, iNtRON, Republic of Korea), phosphatase inhibitor (Sigma, USA), and protease inhibitor (Sigma, USA). The homogenates were centrifuged at 14,000 × g for 20 minutes at 4°C, and the supernatants were collected. Protein concentrations were determined using the Bradford protein assay. For analysis, 25 μg of protein from each sample was mixed with SDS loading buffer, separated through SDS-PAGE, and transferred onto PVDF membranes. Membranes were blocked for 2 hours at room temperature with either 5% skim milk or 3% BSA to minimize nonspecific binding. Subsequently, the membranes were incubated overnight at 4°C with primary antibodies diluted in blocking buffer. The following primary antibodies were used: p-p38 MAPK (1:1000, Cell Signaling), p-eIF2α (1:1000, Cell Signaling), p-PERK (1:1000, Cell Signaling), LAMP2 (1:1000, Invitrogen), LC3B (1:1000, Invitrogen), IL-1β (1:500, Gene Tex), TNF-α (1:500, Abcam), and β-actin (1:10,000, Cell Signaling). After primary antibody incubation, membranes were washed in TBS-T (Bio-Rad) and incubated with secondary antibodies for 1 hour at room temperature. Signals were visualized using a chemiluminescence detection system and captured with a bioimaging instrument (Amersham Imager 680, Marlborough, USA).

### Behavior testing: modified neurological severity score

The modified neurological severity score (mNSS) test was conducted to assess neurological recovery following 7 days of AMX treatment post-TBI. The mNSS provides a quantitative measure of neurological function, with scores ranging from 0 (normal function) to 18 (severe dysfunction). The test evaluates multiple parameters to comprehensively assess neurological deficits: 1) Tail Suspension (3 points)—The rat's ability to be lifted by its tail is assessed, with higher scores indicating increased difficulty or inability to lift. 2) Balance and Posture (3 points)—The rat's ability to maintain proper posture and balance when placed on a flat surface is observed and scored. 3) Sensory Function (3 points)—Sensory responses, including reactions to tactile or pain stimuli, are evaluated. 4) Beam Balance Test (6 points)—The rat's ability to walk on a narrow beam is assessed. Higher scores indicate impaired balance, coordination, or an inability to traverse the beam. 5) Reflexes and Abnormal Movements (4 points)—The presence or absence of reflexes, as well as abnormal behaviors or movements, are recorded and scored. These criteria were used to quantify neurological function and monitor improvements in deficits after AMX treatment. The mNSS test allowed for objectively evaluating the therapeutic effects of AMX on neurological recovery in TBI models 32

### Behavior testing: Morris water maze

The Morris water maze (MWM) test was conducted to evaluate the effects of AMX treatment on spatial learning and memory in rats 8 days after TBI. The MWM is a well-established behavioral test used to assess cognitive function in rodents. A circular water basin (120 cm diameter) was used, with the water depth set to 30 cm and the temperature maintained at 22-26°C. A hidden platform (13 cm diameter) was submerged 1 cm below the water surface to ensure that it was invisible to the rats. Rats underwent training consisting of four trials per day over five consecutive days. In each trial, the rat was released from one of four starting positions, facing the basin wall. The task required the rat to locate the hidden platform within a maximum period of 120 seconds. The following parameters were recorded during the training sessions: 1) escape latency (time taken to reach the platform), 2) distance traveled to locate the platform, and 3) the number of target crossings (times the platform location was crossed). On the final day of testing, a probe trial was conducted to assess memory retention. The hidden platform was removed, and each rat could explore the water basin for 120 seconds. The time spent in the target quadrant (where the platform had been located during training) and the number of crossings over the platform location were recorded. Behavioral data, including escape latency, distance traveled, target crossings, and time spent in the target quadrant, were tracked and analyzed using smart video-tracking software (Panlab, Carrer de l'Energia, Spain). These metrics were used to quantify cognitive impairment and memory function following TBI and AMX treatment 33.

### Statistical analysis

Data were presented as mean ± SD. Statistical analyses were performed using the Mann-Whitney U test or Kruskal-Wallis test for group comparisons, depending on the number of groups analyzed. Behavioral data were analyzed using ANOVA to assess differences between treatment conditions. To ensure objectivity, all analyses were conducted by investigators blinded to the treatment conditions.

## Results

### AMX exhibits PDE-inhibiting activity and restores lysosomal function by increasing cAMP levels

**Figure 1:**
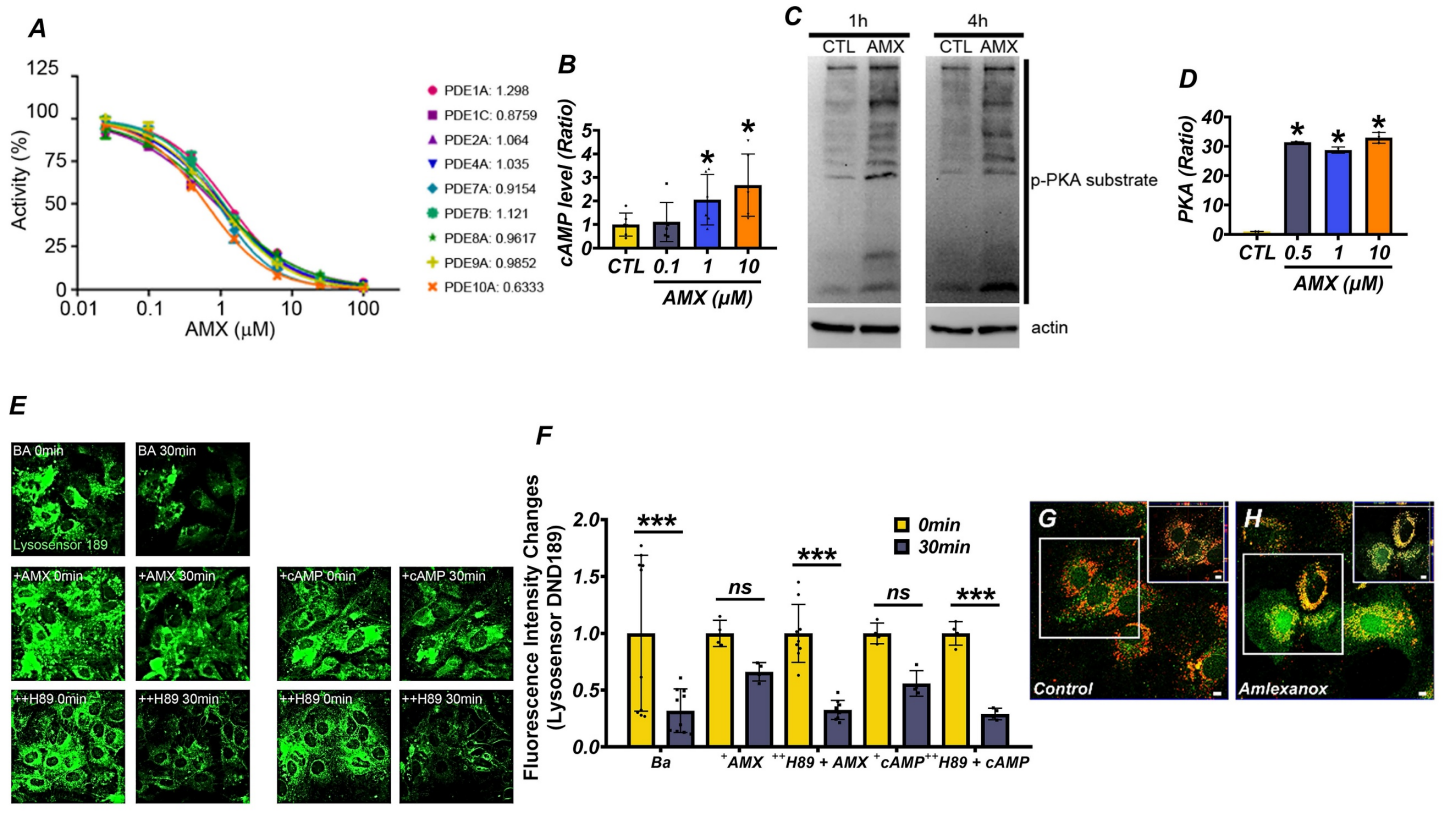
** AMX has PDE-inhibiting capacity and reduces lysosomal dysfunction via increasing cAMP levels. (A)** The IC50 of AMX for various PDE isoforms. **(B)** cAMP levels (pmol/ml) in cultured astrocytes, demonstrating that 1-hour treatment with AMX at concentrations ranging from 0.1 to 10 µM increases cAMP levels (Mean ± SD; # P < 0.05, Kruskal-Wallis test, Bonferroni post hoc: χ² = 9.967, df = 3, p = 0.019). **(C)** Western blotting results, showing levels of p-PKA (pmol/ml) substrate and actin in astrocytes (control, CTL) treated with 10 µM AMX for 1 and 4 hours. **(D)** PKA activity (pmol/ml) in test tubes, indicating that AMX activates PKA within the concentration range of 0.1-10 µM (Mean ± SD; # P < 0.05, Kruskal-Wallis test, Bonferroni post hoc: χ² = 14.119, df = 3, p = 0.003). **(E)** DND-189 fluorescence in astrocytes before (control, CTL) and after 1-hour exposure to bafilomycin A1 (BafA1, BA), BafA1 combined with cAMP (+cAMP), or BafA1 combined with AMX (+AMX) in the absence or presence of H-89 (++H89), a PKA inhibitor. **(F)** Bar graph of DND-189 fluorescence intensity changes (Mean ± SD; # P < 0.05, repeated measures ANOVA Column Factor * group: F = 41.28, p = 0.002, measures Welch's two-sample t-tests between the Ba control and each treatment group: at 30 min, Ba+AMX (P = 0.0005) and Ba+cAMP (P = 0.016) differed significantly from Ba, whereas neither Ba+AMX+H89 (P = 0.92) nor Ba+cAMP+H89 (P = 0.67) showed a significant difference).** (G, H)** Fluorescence photomicrographs of astrocytes stained with FluoZin-3 (green) and lysotracker DND-99 (red), demonstrating increased zinc levels in lysosomes following exposure to AMX.

To evaluate the potential of AMX as a PDE inhibitor, we tested its activity against nine PDE isoforms, namely PDE1A, PDE1C, PDE2A, PDE4A, PDE7A, PDE7B, PDE8A, PDE9A, and PDE10A, all of which hydrolyze cAMP or cyclic guanosine monophosphate (cGMP). Using the PDELight™ HTS cAMP Phosphodiesterase Assay, AMX demonstrated significant inhibitory effects on all tested isoforms, with a half-maximal inhibitory concentration (IC50) of approximately 1 μM (Figure 1A) (P < 0.05). Recognizing the pivotal role of cAMP in activating downstream signaling pathways such as the PKA pathway, we next examined the effects of AMX on intracellular cAMP levels and PKA activity in cultured cortical astrocytes. Treatment with AMX significantly increased intracellular cAMP levels in a concentration-dependent manner, particularly at 1 μM and 10 μM (Figure 1B) (P < 0.05). This increase in cAMP corresponded to increased p-PKA levels (Figure 1C) (P < 0.05), indicating enhanced PKA activity (Figure 1D) (P < 0.05). Interestingly, however, the increase in p-PKA levels was not strictly proportional to the concentration-dependent increase in cAMP. This discrepancy may reflect the compartmentalized nature of intracellular cAMP signaling, where global increases in cAMP do not uniformly activate PKA across all cellular compartments. PKA activation typically requires threshold cAMP concentrations within discrete microdomains to initiate phosphorylation events. Additionally, negative feedback mechanisms including the cAMP-induced activation of PDEs or the preferential engagement of alternative cAMP-dependent pathways such as Epac may also contribute to this differential response between total cAMP levels and PKA activation. To explore the downstream functional consequences of PKA activation, we assessed lysosomal pH changes using DND-189, a pH-sensitive fluorescent dye selective for lysosomes. Under normal conditions, lysosomes maintained an acidic pH, while treatment with 100 nM of bafilomycin A1 (BafA1), a lysosomal proton pump inhibitor, markedly alkalinized lysosomes. Treatment with 10 μM of AMX or 300 μM of cAMP effectively reversed BafA1-induced alkalization, restoring lysosomal acidity. Notably, co-treatment with the PKA inhibitor H-89 abolished the lysosomal acidification effect of AMX, with fluorescence levels returning to those seen in BafA1-treated astrocytes (Figure 1E, F) (P < 0.05). Given the importance of lysosomal zinc in regulating lysosomal pH, we further investigated the effects of AMX on lysosomal zinc concentrations. Using FluoZin-3, a zinc-sensitive fluorescent dye, together with LysoTracker for lysosome localization, we observed a significant increase in zinc fluorescence within lysosomes following treatment with 10 μM of AMX for 30 minutes (Figure S1A). Merged images confirmed that the increased zinc signals were specifically localized to lysosomes. In addition, fluorescence photomicrographs of astrocytes co-stained with FluoZin-3 (green) and LysoTracker DND-99 (red) demonstrated that AMX treatment markedly increased lysosomal zinc levels, as shown by enhanced green fluorescence colocalized with lysosomal compartments (Figure 1G, H). Taken together, these findings demonstrate that AMX inhibits multiple PDE isoforms, increases intracellular cAMP levels, and restores lysosomal acidity through the cAMP-PKA signaling pathway. The observed divergence between cAMP levels and PKA activation underscores the complexity of intracellular cAMP signaling dynamics and suggests the involvement of spatially restricted cAMP pools and feedback regulatory mechanisms. Furthermore, the modulation of lysosomal zinc levels by AMX, as shown in Figure 1G, H, highlights a novel mechanism through which it alleviates lysosomal dysfunction.

### AMX enhances cell viability and alleviates lysosomal dysfunction by increasing cAMP levels after mechanical scratch injury

**Figure 2:**
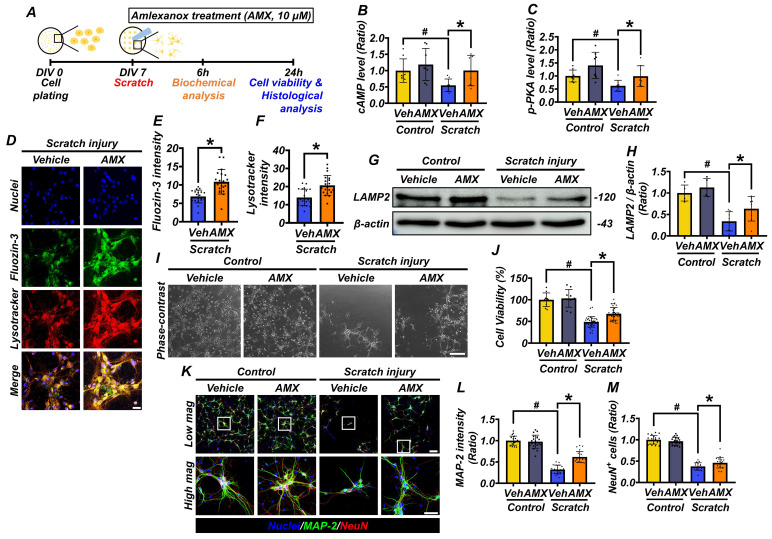
** AMX increases cell viability and alleviates lysosomal dysfunction by increasing cAMP levels after mechanical scratch injury. (A)** Experimental timeline: AMX was applied immediately after the scratch assay. cAMP levels (pmol/ml) were measured at 6 hours and cell viability at 24 hours after the scratch.** (B)** Bar graph shows cAMP levels quantified using ELISA (n=8/group). # P < 0.05 vs. sham; * P < 0.05 vs. vehicle-treated TBI (Mean ± SD; Kruskal-Wallis test, Bonferroni post hoc: χ² = 10.462, df = 3, p = 0.015). **(C)** Bar graph shows p-PKA levels (pmol/ml) quantified using ELISA (n=8/group). # P < 0.05 vs. sham; * P < 0.05 vs. vehicle-treated TBI (Mean ± SD; Kruskal-Wallis test, Bonferroni post hoc: χ² = 13.377, df = 3, p = 0.004).** (D)** Immunofluorescence images of primary hippocampal neurons co-stained with Fluozin-3 (green) and Lysotracker (red) 24 hours after scratch. Scale bar: 50 μm. **(E, F)** Bar graphs of Fluozin-3 **(E)** and Lysotracker **(F)** intensity (n=20/group for TBI) (Mean ± SD; Mann-Whitney U: Fluozin-3: z = 4.247, p < 0.001; Lysotracker: z = 3.327, p = 0.001).** (G)** Western blot of LAMP2, 24 hours after scratch.** (H)** LAMP2 expression quantified (n=5/sham group, n=8/TBI group). # P < 0.05 vs. sham; * P < 0.05 vs. vehicle-treated TBI (Mean ± SD; Kruskal-Wallis, Bonferroni: χ² = 17.216, df = 3, p = 0.001). **(I)** Representative neuron images after scratch. Scale bar: 100 μm.** (J)** MTT assay for cell viability (n=10/sham group, n=36/TBI group). # P < 0.05 vs. sham; * P < 0.05 vs. vehicle-treated TBI (Mean ± SD; Kruskal-Wallis, Bonferroni: χ² = 57.885, df = 3, p < 0.001).** (K)** Co-staining of MAP2 (green) and NeuN (red) in neurons, 24 hours after scratch. Scale bar: 50 μm.** (L)** MAP2 intensity quantification (n=20/group). # P < 0.05 vs. sham; * P < 0.05 vs. vehicle-treated TBI (Mean ± SD; Kruskal-Wallis, Bonferroni: χ² = 63.97, df = 3, p < 0.001).** (M)** NeuN-positive cell count (n=20/group). # P < 0.05 vs. sham; * P < 0.05 vs. vehicle-treated TBI (Mean ± SD; Kruskal-Wallis, Bonferroni: χ² = 61.086, df = 3, p < 0.001).

To investigate the therapeutic potential of AMX, primary hippocampal neurons were cultured from embryonic day 18 (E18) rats. On the seventh day *in vitro* (DIV 7), a scratch assay was performed to simulate mechanical injury. Following the scratch injury, both control- and scratch-treated cells were exposed to AMX, and analyses were conducted at 6 and 24 hours post-injury (Figure 2A). AMX (10 μM) was administered immediately following the scratch injury, and intracellular cAMP and p-PKA levels were quantified via ELISA at 6 hours post-injury. This early time point was selected to assess the alterations in cAMP and p-PKA, which function as upstream signaling molecules in the pathway. Scratched neurons exhibited significant reductions in both cAMP and p-PKA levels compared to controls. Notably, treatment with AMX restored cAMP and p-PKA levels in scratched neurons to values comparable to those observed in the control group, suggesting its ability to the counteract injury-induced depletion of these signaling molecules (Figure 2B, C) (P< 0.05).

Moreover, AMX treatment enhanced neuroprotection in neurons by increasing the phosphorylation of PKA, a key downstream effector of the cAMP signaling pathway (Figure S2E-J) (P< 0.05). To explore the underlying mechanisms, PDE4 expression was analyzed through both immunocytochemistry and Western blotting. PDE4 levels were significantly reduced in the scratch-AMX group compared to the scratch-vehicle group, suggesting that AMX exerts its effects by inhibiting PDE4 activity (Figure S2A-D), (P< 0.05). Lysosomal function was evaluated by measuring lysosomal zinc levels and LAMP2 expression. Immunofluorescence staining using FluoZin-3 (a zinc-sensitive dye) and LysoTracker (a lysosome-specific dye) revealed markedly reduced lysosomal zinc levels in scratched neurons. AMX treatment restored lysosomal zinc levels to near-normal levels (Figures 2D-F) (P< 0.05). As part of our experimental controls, we conducted identical staining procedures on the control group under the same imaging conditions. However, no detectable fluorescence signals for either FluoZin-3 or LysoTracker were observed in the control samples, making it technically infeasible to capture representative control images. This observation is consistent with previous reports indicating that, under physiological conditions, intracellular free zinc concentrations and lysosomal activity remain at basal levels, typically below the detection threshold of fluorescence-based assays 14, 34, 35. Western blot analysis further showed that LAMP2 expression, though diminished in the scratch-vehicle group, was significantly preserved in the scratch-AMX group, indicating improved lysosomal integrity (Figures 2G, H) (P< 0.05). At 24 hours post-scratch, cell viability was assessed using an MTT assay. Neuronal viability was significantly reduced in the scratch-vehicle group compared with sham controls. AMX treatment substantially improved cell viability in scratched neurons (Figure 2I, J) (P< 0.05). Further analysis of neuronal morphology through immunocytochemistry revealed that AMX mitigated scratch-induced dendritic damage. Increased MAP2 fluorescence intensity indicated reduced dendritic loss in AMX-treated neurons. Additionally, the number of NeuN-positive neurons was significantly higher in the AMX-treated group, demonstrating enhanced neuronal survival (Figures 2K-M) (P< 0.05). These findings demonstrate that AMX restores cAMP levels, alleviates lysosomal dysfunction, preserves lysosomal integrity, reduces dendritic loss, and enhances neuronal survival following mechanical injury. These results underscore the protective role of AMX in maintaining cell viability and lysosomal function under conditions of mechanical stress, highlighting its therapeutic potential for mitigating injury-induced cellular damage.

### AMX mitigates neuronal degeneration by increasing cAMP levels and regulating intracellular processes

**Figure 3:**
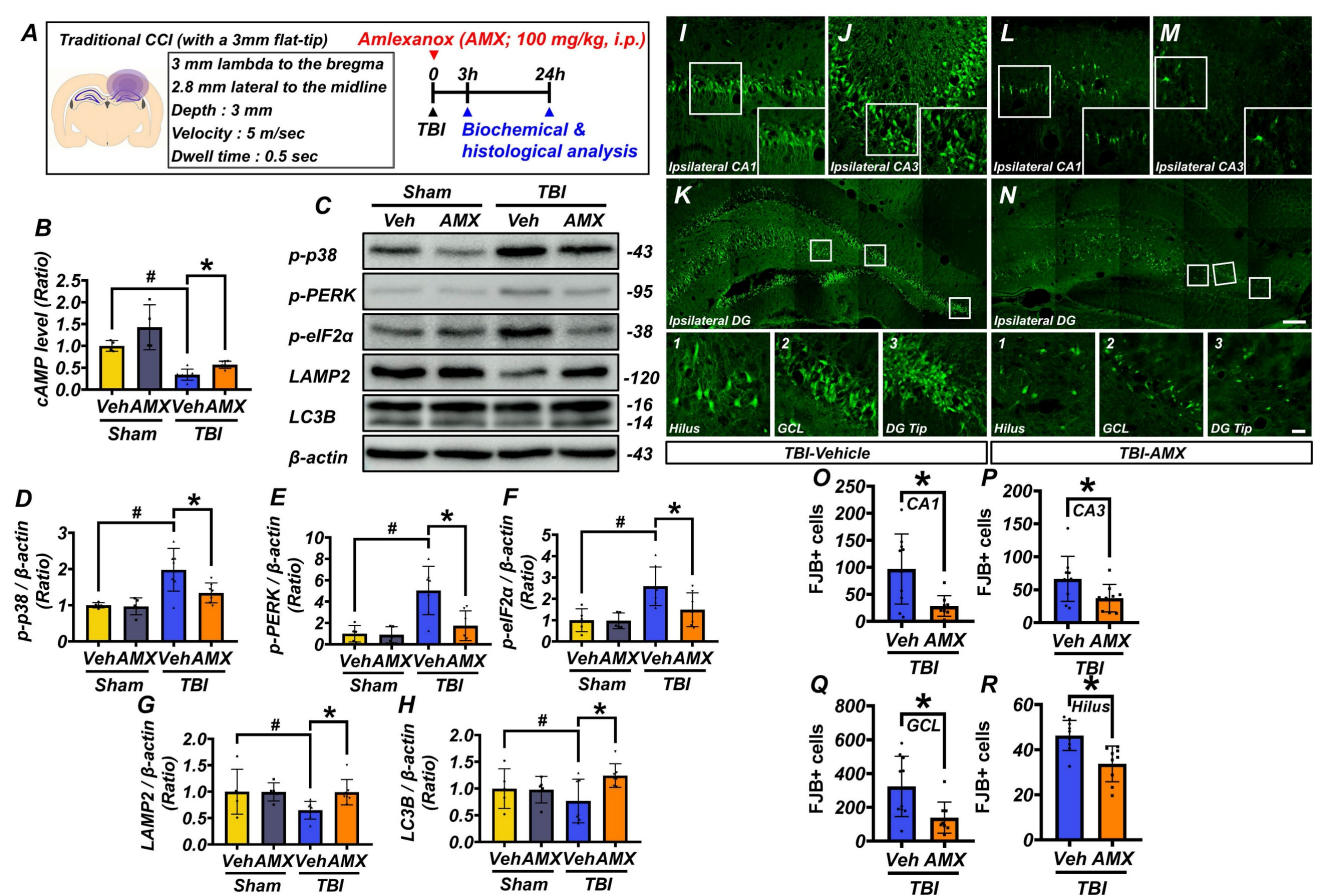
** AMX relieves neuronal degeneration by increasing cAMP levels and regulating intracellular activity. (A)** Experimental timeline: AMX was administered immediately after TBI, with rats sacrificed at either 3 or 24 hours. **(B)** Bar graph showing cAMP levels measured using ELISA (n=4/sham group, n=7/TBI group). # P < 0.05 vs. sham; * P < 0.05 vs. vehicle-treated TBI (Mean ± SD; Kruskal-Wallis, Bonferroni post hoc: χ² = 17.646, df = 3, p = 0.001). **(C)** Western blot results 24 hours after TBI assessing p38 MAPK phosphorylation (p-p38), eIF2α phosphorylation (p-eIF2α), PERK phosphorylation (p-PERK), LAMP2, and LC3B in the hippocampus.** (D-H)** Bar graphs quantifying protein expression levels of p-p38 **(D)**, p-PERK **(E)**, p-eIF2α **(F)**, LAMP2 **(G)**, and LC3B **(H)** in the hippocampus (n=5/sham group, n=7/TBI group). # P < 0.05 vs. sham; * P < 0.05 vs. vehicle-treated TBI (Mean ± SD; Kruskal-Wallis, Bonferroni post hoc: p-p38: χ² = 10.569, df = 3, p = 0.014; p-eIF2α: χ² = 11.445, df = 3, p = 0.01; p-PERK: χ² = 11.145, df = 3, p = 0.011; LAMP2: χ² = 9.682, df = 3, p = 0.021; LC3B: χ² = 8.369, df = 3, p = 0.039).** (I-N)** Immunofluorescence images of ipsilateral hippocampal CA1 **(I, L)**, CA3 **(J, M)**, and dentate gyrus **(K, N)** regions, stained with FJB for degenerating neurons, 24 hours after TBI. Scale bar: 100 μm. **(O-R)** Bar graphs quantify FJB-positive cells in each hippocampal regions CA1 **(O)**, CA3 **(P)**, GCL **(Q)**, hilus **(R)** (n=9-10/TBI group). * P < 0.05 vs. vehicle-treated TBI (Mean ± SD; Mann-Whitney U: CA1: z = 2.205, p = 0.027; CA3: z = 2.123, p = 0.035; GCL: z = 2.531, p = 0.010; hilus: z = 3.063, p = 0.001).

This study examined the effects of AMX on neuronal death in the hippocampus following TBI. Rats were administered AMX immediately after TBI or a sham procedure, and hippocampal tissues were collected for analysis at 3 and 24 hours post-injury (Figure 3A). At 3 hours post-TBI, hippocampal cAMP levels, measured using ELISA, were significantly reduced in the TBI-vehicle group compared to sham controls. However, AMX treatment restored cAMP levels to values comparable to sham controls, suggesting that AMX counteracts TBI-induced cAMP depletion (Figure 3B) (P< 0.05). To assess cellular stress, including ER stress, immunoblotting was performed on hippocampal tissue at 24 hours post-TBI (Figure 3C). In sham animals, phosphorylation levels of p38 MAPK, PERK, and eIF2α were not significantly affected by AMX treatment. In contrast, in the TBI groups, the phosphorylation of these markers was significantly increase in the TBI-vehicle group, indicating increased cellular and ER stress. AMX treatment significantly reduced the phosphorylation levels of p38 MAPK, PERK, and eIF2α compared to the TBI-vehicle group, suggesting that AMX alleviates TBI-induced cellular and ER stress (Figures 3D-F) (P< 0.05). Markers of lysosomal integrity (LAMP2) and autophagy (LC3B) were evaluated to determine the impacts of TBI on these processes. In the TBI-vehicle group, LAMP2 and LC3B levels were significantly reduced, indicating lysosomal dysfunction and impaired autophagy. AMX treatment preserved LAMP2 and LC3B expression, suggesting that it restores lysosomal function and supports autophagic processes disrupted by TBI (Figures 3G, H), (P< 0.05). Neuronal degeneration in the hippocampus was assessed using FJB staining at 24 hours post-TBI. As expected and consistent with previous reports, no FJB-positive neurons were detected in the sham group. Therefore, representative images and quantification for the sham group were omitted from Figure 3I-R to avoid presenting redundant data. Analyses were, therefore, focused on TBI groups in which neurodegeneration was detectable. In the TBI-vehicle group, widespread neuronal degeneration was observed in the ipsilateral hippocampal regions, including CA1, CA3, the GCL of the dentate gyrus, and the hilus. AMX treatment significantly reduced the number of FJB-positive degenerating neurons in these regions compared to the TBI-vehicle group (Figures 3I-R) (P< 0.05). These results demonstrate that AMX mitigates TBI-induced neuronal degeneration by restoring cAMP levels, reducing cellular and ER stress, and preserving lysosomal and autophagic functions. The findings highlight AMX's therapeutic potential in reducing neuronal damage and supporting recovery after TBI.

### AMX reduces oxidative stress and modulates glial cell activation to alleviate neuroinflammation

**Figure 4:**
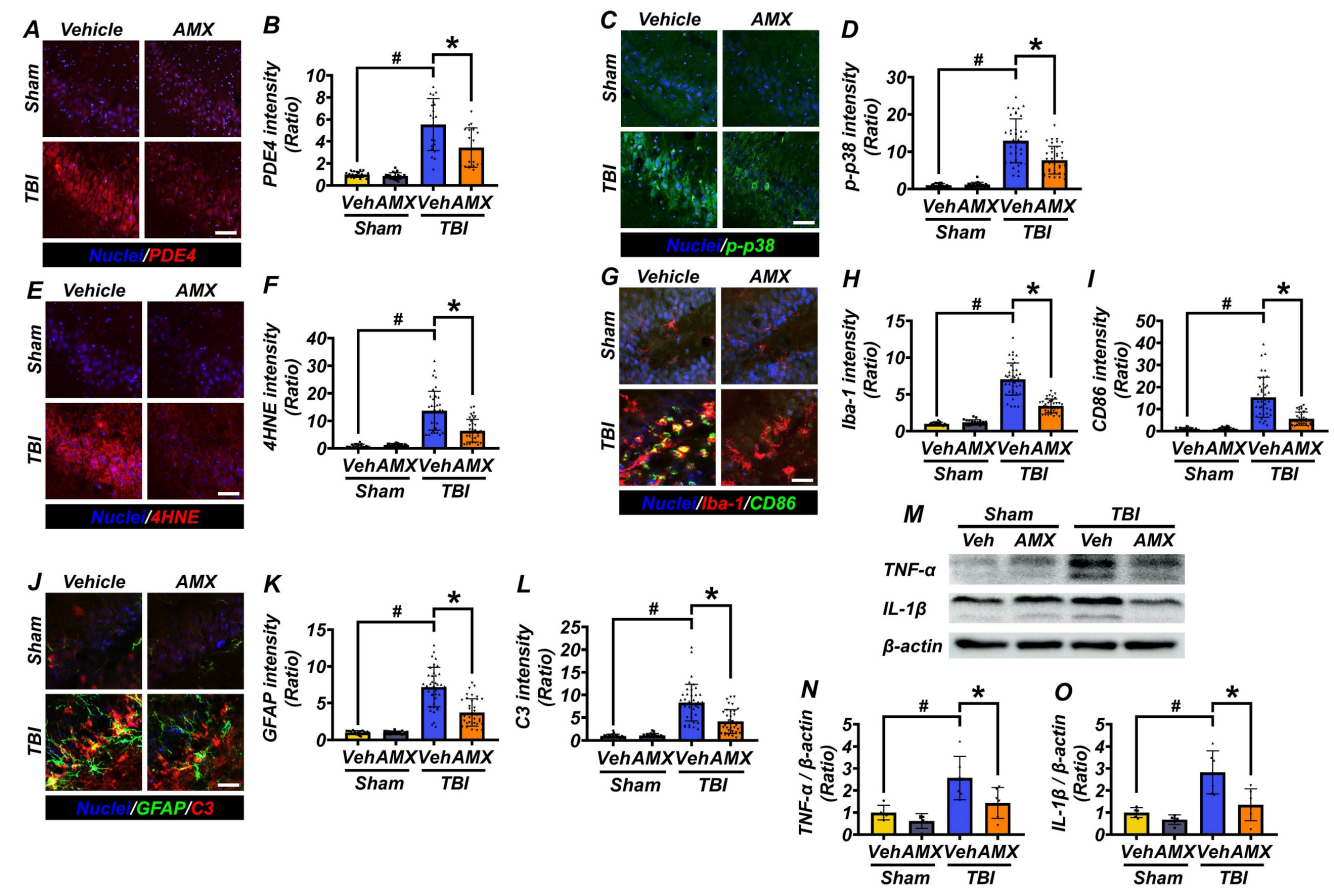
** AMX reduces PDE4 expression, cellular stress, and inflammation after TBI. (A)** Images of PDE4 staining (red) in hippocampal regions, 3 hours after TBI. Nuclei are DAPI-stained (blue). Scale bar = 100 μm. **(B)** Bar graph of PDE4 intensity (n=5/group). # P < 0.05 vs. sham; * P < 0.05 vs. vehicle-treated TBI (Mean ± SD; Kruskal-Wallis, Bonferroni: CA1, CA3, GCL, hilus all χ² > 15, df = 3, p ≤ 0.002). **(C)** Immunofluorescence images of the hippocampus (CA1, CA3, GCL, hilus) show p-p38 (green) 24 hours after TBI. Nuclei are counterstained with DAPI (blue). Scale bar = 100 μm. **(D)** Bar graph quantifies p-p38 intensity in each region (n=5/sham, n=9-10/TBI). # P < 0.05 vs. sham; * P < 0.05 vs. vehicle-treated TBI (Mean ± SD; Kruskal-Wallis, Bonferroni: CA1, CA3, GCL, hilus all χ² > 21, df = 3, p < 0.001). **(E)** 4HNE staining (red) of lipid peroxidation after TBI. Nuclei are DAPI-stained (blue). Scale bar = 100 μm. **(F)** Bar graph of 4HNE intensity (n=5/sham, n=9-10/TBI). # P < 0.05 vs. sham; * P < 0.05 vs. vehicle-treated TBI (Mean ± SD; Kruskal-Wallis, Bonferroni: CA1, CA3, GCL, hilus all χ² > 21, df = 3, p < 0.001). **(G)** Images of CA1, CA3, GCL, and hilus with Iba-1 (red) and CD86 (green) staining for microglia, with merged images included 24 hours after TBI. Nuclei are DAPI-stained (blue). Scale bar = 100 μm. **(H, I)** Bar graphs quantifying Iba-1 **(H)** and CD86 **(I)** intensity (n=5/sham, n=9-10/TBI). # P < 0.05 vs. sham; * P < 0.05 vs. vehicle-treated TBI (Mean ± SD; Kruskal-Wallis, Bonferroni: Iba-1 and CD86: CA1, CA3, GCL, and hilus all χ² > 19, df = 3, p < 0.001). **(J)** Images of CA1, CA3, GCL, and hilus with GFAP (green) and C3 (red) staining for astrocytes, 24 hours after TBI. Nuclei are DAPI-stained (blue). Scale bar = 100 μm. **(K, L)** Bar graphs of GFAP **(K)** and C3 **(L)** intensity (n=5/sham, n=9-10/TBI). # P < 0.05 vs. sham; * P < 0.05 vs. vehicle-treated TBI (Mean ± SD; Kruskal-Wallis, Bonferroni: GFAP and C3: CA1, CA3, GCL, hilus all χ² > 19, df = 3, p < 0.001). **(M)** Western blot results 24 hours after TBI assessing TNF-α and IL-1β (Interleukin-1 beta) in the hippocampus. **(N, O)** Bar graphs quantifying protein expression levels of TNF-α **(N)**, IL-1β **(O)** in the hippocampus (n=5/sham group, n=6/TBI group). # P < 0.05 vs. sham; * P < 0.05 vs. vehicle-treated TBI (Mean ± SD; Kruskal-Wallis, Bonferroni: TNF-α: χ² = 12.526, df = 3, p = 0.006; IL-1β: χ² = 13.732, df = 3, p = 0.03).

To evaluate the anti-inflammatory effects of AMX, hippocampal tissue was analyzed for markers of oxidative stress, glial activation, and neuroinflammation following TBI. At 3 hours post-TBI, hippocampal PDE4 expression, assessed via immunofluorescence staining, was significantly increased in the TBI-vehicle group compared with sham controls. AMX treatment markedly reduced PDE4 expression in the hippocampi of TBI rats, lowering it to levels closer to those observed in sham controls (Figures 4A, B) (Figure S3A, B) (P< 0.05). At 24 hours post-TBI, p-p38, a marker of cellular stress, was evaluated in hippocampal regions. Immunofluorescence analysis revealed significantly reduced p-p38 levels in the TBI-AMX group compared with the TBI-vehicle group, indicating decreased stress signaling (Figures 4C, D) (Figure S4A, B) (P< 0.05). Lipid peroxidation, a hallmark of oxidative stress, was examined using 4HNE staining. Minimal 4HNE staining was observed in sham groups, whereas the TBI-vehicle group exhibited markedly increased oxidative stress in the hippocampus. Treatment with AMX significantly reduced 4HNE staining in the hippocampal regions, demonstrating its capacity to mitigate oxidative damage (Figures 4E, F) (Figure S4C, D) (P< 0.05). To assess the impact of AMX on microglial activation and polarization, double immunofluorescence staining for Iba-1 (a general microglial marker) and CD86 (an M1 polarization marker) was performed 36. The TBI-vehicle group showed widespread microglial activation with increased M1-polarized microglia in the hippocampus. AMX treatment significantly reduced both microglial activation and M1 polarization, as evidenced by decreased Iba-1 and CD86 staining in the hippocampal regions (Figures 4G-I) (Figure S5A-F) (P< 0.05). Astrocyte activation was evaluated using double immunofluorescence staining for GFAP (a marker of activated astrocytes) and C3 (a marker associated with neuroinflammation). In the TBI-vehicle group, astrocytes exhibited hypertrophic morphology with thickened processes, indicative of significant activation. AMX treatment reduced astrocytic activation, as demonstrated by lower GFAP and C3 intensity and fewer hypertrophic astrocytic processes occurring in the hippocampus (Figures 4J-L) (Figure S6A-F) (P< 0.05). Additionally, we performed Western blot analysis to assess changes in the expression of the inflammatory cytokines TNF-α and IL-1β in the hippocampus following TBI. The results revealed significant increases in TNF-α and IL-1β levels in the TBI-vehicle group, whereas AMX treatment markedly reduced the expression of both cytokines (Figures 4M-O) (P< 0.05). These findings demonstrate that AMX mitigates TBI-induced oxidative stress, reduces the pro-inflammatory activity of glial cells, and alleviates neuroinflammation in the hippocampus. By reducing cellular stress and modulating the activity of microglia and astrocytes, AMX shows promise as a therapeutic intervention for TBI.

### AMX mitigates TBI-induced neurological impairment, cognitive deficits, and delayed neuronal loss

**Figure 5:**
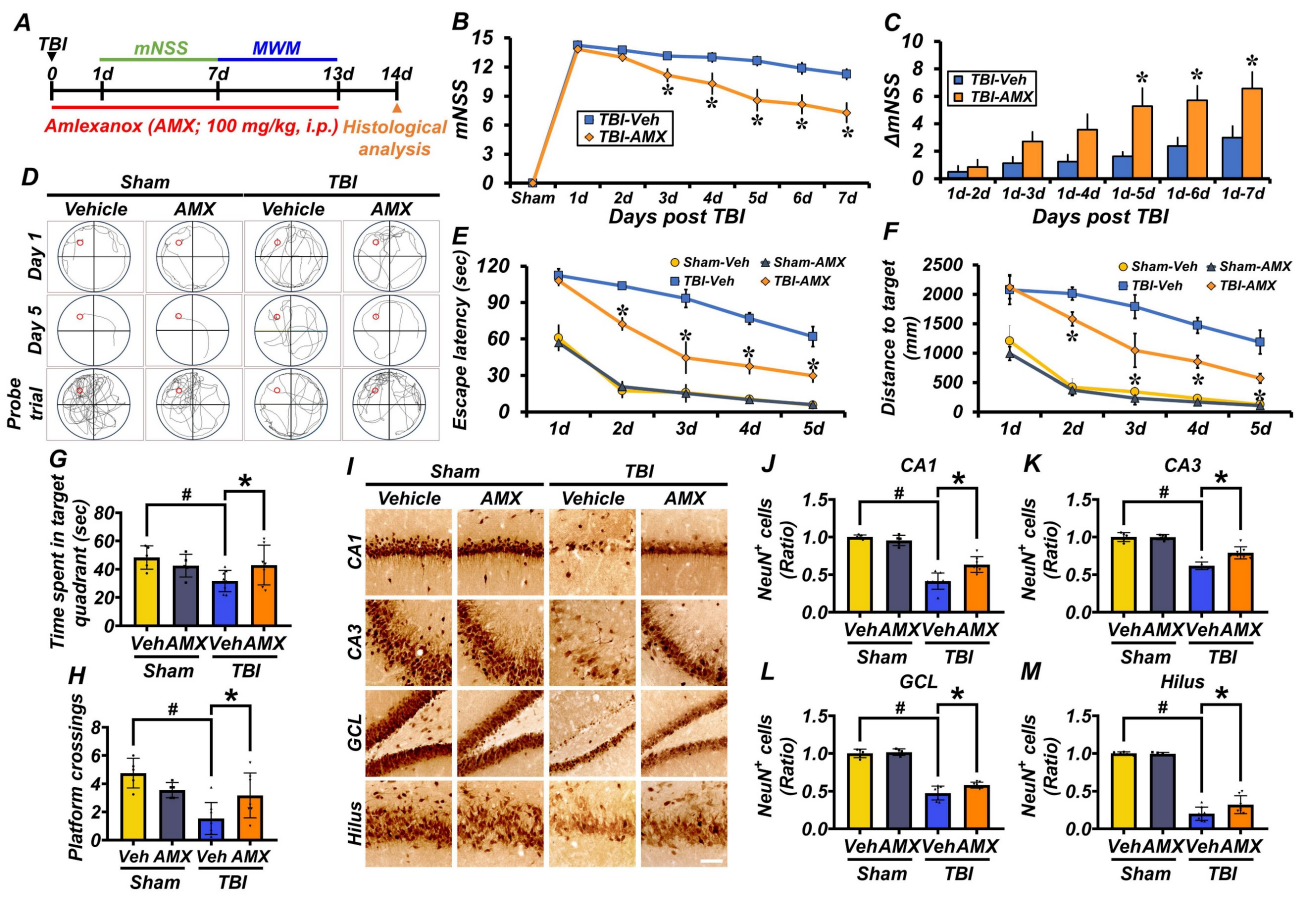
** AMX ameliorates TBI-induced neurological impairment and cognitive deficits and delays neuronal loss. (A)** Experimental design involved daily intraperitoneal AMX administration for 14 days, with rats sacrificed two weeks after TBI.** (B)** The mNSS was assessed from days 1 to 7 after TBI, ranging from 0 (all tests passed) to 18 (all tests failed). * P < 0.05 vs. vehicle-treated TBI group (Mean ± SD; repeated measures ANOVA: F = 24.881, p < 0.001; group: F = 15.604, p = 0.002; time * group: F = 4.689, p < 0.001).** (C)** ΔmNSS measured between day 1 and subsequent time points (n=5/sham, n=7-8/TBI). * P < 0.05 vs. vehicle-treated TBI (ANOVA).** (D)** Representative MWM swim tracking images.** (E, F)** Morris water maze (MWM) tests conducted days 8-12 after TBI, assessing platform arrival time **(E)** and distance **(F)** (Mean ± SD; repeated measures ANOVA: escape latency: F = 31.62, p < 0.001; group: F = 13.26, p < 0.001; time * group: F = 1.456, p = 0.158; distance: F = 31.714, p < 0.001; group: F = 11.015, p < 0.001; time * group: F = 1.697, p = 0.082).** (G, H)** MWM probe trial on day 13 after TBI, evaluating time in target quadrant **(G)** and platform crossings **(H)** (n=5/sham, n=7-8/TBI). # P < 0.05 vs. sham; * P < 0.05 vs. vehicle-treated TBI (Mean ± SD; Kruskal-Wallis, Bonferroni: time in quadrant: χ² = 8.273, p = 0.041; platform crossings: χ² = 12.919, p = 0.005).** (I)** Hippocampal regions (CA1, CA3, GCL, hilus) stained with NeuN, 2 weeks after TBI. Scale bar = 100 μm.** (J-M)** Quantification of NeuN-positive cells in each hippocampal regions CA1 **(J)**, CA3 **(K)**, GCL **(L)**, hilus **(M)** (n=5/sham, n=7-8/TBI). # P < 0.05 vs. sham; * P < 0.05 vs. vehicle-treated TBI (Mean ± SD; Kruskal-Wallis, Bonferroni: CA1: χ² = 20.8; CA3: χ² = 20.309; GCL: χ² = 19.139; hilus: χ² = 18.941; all df = 3, p < 0.001).

This study evaluated the neuroprotective effects of AMX on behavioral impairments, cognitive deficits, and neuronal damage induced by TBI. Behavioral outcomes were assessed using the mNSS and MWM tests. The mNSS test, conducted starting 24 hours post-TBI, assessed motor, sensory, balance, and reflex functions. Rats in the TBI-vehicle group showed significant behavioral impairments, as indicated by persistently high mNSS scores and minimal improvement in ΔmNSS over time. In contrast, the TBI-AMX group demonstrated significant neurological recovery, reflected by lower mNSS scores and greater improvements in ΔmNSS compared with the vehicle-treated group (Figures 5B, C) (P< 0.05). The MWM test was conducted between days 8 and 12 post-TBI to evaluate spatial learning and memory. Rats in the TBI-vehicle group exhibited marked deficits, including prolonged escape latency, increased distances traveled to the hidden platform, reduced time spent in the target quadrant, and fewer platform crossings during the probe trial. AMX treatment significantly improved cognitive performance, as evidenced by shorter escape latency, reduced target distances, increased time spent in the target quadrant, and more frequent platform crossings compared with the TBI-vehicle group (Figures 5D-H) (P< 0.05). To assess long-term neuroprotection, hippocampal neuronal viability was evaluated at 2 weeks post-TBI using NeuN staining. Sham groups displayed abundant NeuN-positive cells in hippocampal regions, whereas the TBI-vehicle group exhibited significantly reduced NeuN-positive neurons. AMX treatment preserved neuronal viability, as shown by higher NeuN-positive cell counts in the CA1, CA3, GCL, and hilus regions of the hippocampus compared with the TBI-vehicle group (Figures 5I-M) (P< 0.05). The integrity of the BBB was assessed by measuring IgG extravasation in the ipsilateral hippocampus at 24 hours and 2 weeks post-TBI. The TBI-vehicle group displayed substantial IgG leakage, indicating significant BBB disruption. However, AMX treatment significantly reduced IgG extravasation, suggesting that it helped to maintain BBB integrity (Figure S7A-D). These findings demonstrate that AMX treatment mitigates TBI-induced behavioral and cognitive impairments, preserves neuronal viability in the hippocampus, and maintains BBB integrity. Collectively, these results highlight the therapeutic potential of AMX for promoting recovery and neuroprotection after TBI.

## Discussion

**Figure 6:**
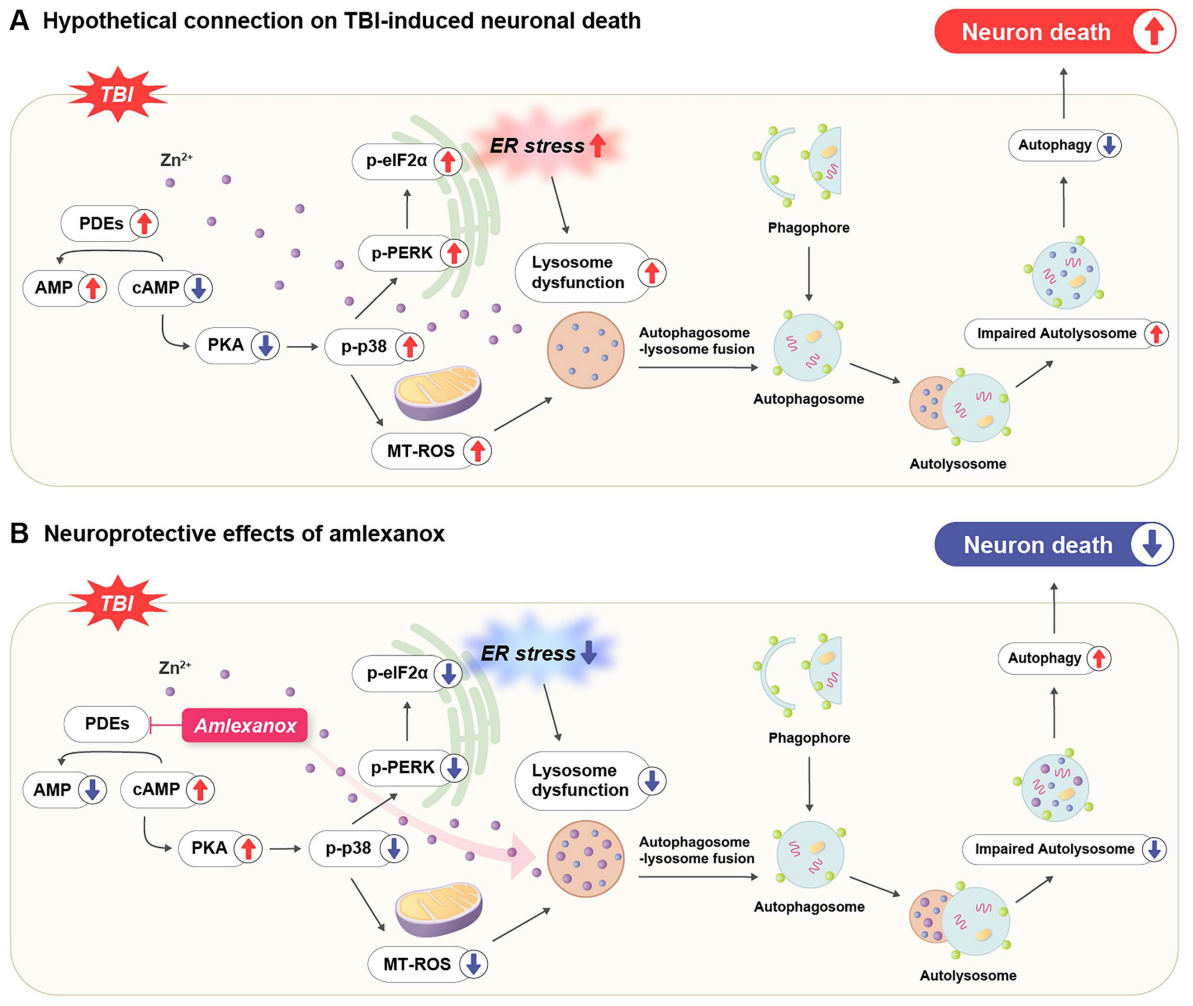
** A schematic illustration of the proposed mechanism through which amlexanox attenuates TBI-induced neuronal death. (A) Pathophysiological cascade following TBI:** TBI leads to an excessive influx of intracellular Zn²⁺, which in turn activates PDE enzymes. Elevated PDE activity decreases intracellular cAMP levels, resulting in reduced activation of PKA. Diminished PKA signaling contributes to the increased phosphorylation of p38 MAPK and elevated mitochondrial reactive oxygen species (MT-ROS) production, ultimately leading to mitochondrial dysfunction. Concurrently, TBI induces ER stress through activating the PERK pathway and phosphorylation of eIF2α, impairing lysosomal function. This disruption in lysosomal activity inhibits autophagosome-lysosome fusion, causing autolysosome accumulation and inefficient autophagic flux, which together exacerbate neuronal apoptosis and cell death. **(B) Neuroprotective mechanism of AMX following TBI:** Post-injury administration of AMX inhibits PDE activity, resulting in increased intracellular cAMP levels and reactivated PKA signaling. Activated PKA suppresses p-p38 MAPK phosphorylation and reduces MT-ROS generation, thereby stabilizing mitochondrial function. In parallel, AMX attenuates ER stress by downregulating the p-PERK and p-eIF2α pathways, thereby restoring lysosomal integrity. Enhanced lysosomal function promotes autophagosome-lysosome fusion and improves autophagic flux by reducing autolysosome accumulation. These combined effects of AMX alleviate multiple secondary injury mechanisms and contribute to reduced neuronal death following TBI.

TBI triggers a complex cascade of secondary injury mechanisms that amplify the initial mechanical damage. These include oxidative stress, neuroinflammation, lysosomal dysfunction, and impaired autophagy 28, 37, 38. Previous studies have shown that TBI rapidly decreases intracellular cAMP levels in affected brain regions, particularly the cortex and hippocampus, thereby exacerbating neuroinflammation and neuronal vulnerability through suppressing PKA signaling 37. The pharmacological enhancement of cAMP signaling via PDE inhibition has been demonstrated to mitigate these secondary injury processes and improve outcomes in experimental models of TBI, supporting the therapeutic relevance of targeting cyclic nucleotide pathways 37. This provides a strong mechanistic rationale for targeting cyclic nucleotide pathways in TBI therapy 37. In this context, our study provides compelling evidence for the therapeutic potential of AMX, a broad-spectrum PDE inhibitor, in modulating multiple pathophysiological processes involved in TBI. A key finding is that AMX enhances both cAMP and cGMP signaling by inhibiting multiple PDE isoforms, whereas selective inhibitors only target a single isoform 28, 37. This multi-targeted mechanism allows AMX to exert broader neuroprotective effects, including attenuating neuroinflammation and oxidative stress, as well as modulating synaptic plasticity through activating both the PKA and protein kinase G (PKG) pathways. Importantly, unlike conventional PDE4 inhibitors, AMX's ability to inhibit several PDE subtypes may confer a wider therapeutic window and more comprehensive protection against TBI-related neuropathology 20, 21, 28, 37. These findings highlight that AMX is a promising candidate for TBI treatment due to it targeting the cyclic nucleotide signaling network (Figure 6).

PDEs are enzymes that degrade cyclic nucleotides such as cAMP and cGMP, regulating diverse cellular processes 39. Among these, PDE4 is a well-established target in neurological disorders due to its role in neuroinflammation and synaptic plasticity 40, 41. AMX, previously identified as a PDE4 inhibitor, exhibits anti-inflammatory actions by targeting PDE4B in macrophages 28. This study expands upon this by demonstrating that AMX inhibits multiple PDE isoforms, including PDE1, PDE2, PDE7, and PDE8 20, 21. This broader inhibition profile enables AMX to modulate both cAMP and cGMP signaling pathways 28, 42, 43. For example, PDE1 and PDE2 hydrolyze both cyclic nucleotides, while PDE7 and PDE8 are involved in immune regulation and stress response signaling, respectively 44. By preventing nucleotide degradation, AMX enhances the activity of downstream kinases such as PKA and PKG, which are critical for neuronal survival, synaptic plasticity, and vascular function 43. The ability to simultaneously target multiple PDEs distinguishes AMX from selective PDE inhibitors, offering potential advantages in addressing the multifactorial pathology of TBI 28. However, given its relatively modest potency (approximately 1 μM) in purified enzyme assays, the possibility of additional off-target effects cannot be excluded. Notably, previous studies have identified AMX as a dual inhibitor of TANK-binding kinase 1 (TBK1) and IκB kinase ε (IKK-ε), both of which are involved in neuroinflammatory and neurodegenerative pathways 45. These additional molecular targets may contribute to the broader neuroprotective effects observed with AMX treatment. While our current findings highlight that cAMP-mediated pathways are the primary mechanism, it is plausible that these off-target effects may also contribute to the observed neuroprotection. Further studies are needed to fully delineate these overlapping mechanisms and evaluate their therapeutic relevance in TBI.

Additionally, the demonstrated reduction in oxidative and ER stress markers following AMX treatment supports the notion of a mechanistic role for cyclic nucleotide modulation in maintaining mitochondrial and ER integrity 46. TBI-induced oxidative stress originates predominantly from mitochondrial dysfunction and NADPH oxidase activation, which amplify lipid peroxidation and DNA damage 47, 48. By enhancing cAMP-PKA signaling, AMX may promote the phosphorylation of mitochondrial proteins involved in respiratory chain stability and ROS detoxification, thus breaking the vicious cycle between oxidative and ER stress 49-53. This interpretation is supported by reduced 4-HNE accumulation and decreased PERK-eIF2α phosphorylation in AMX-treated animals, indicating relief from ER overload and associated apoptotic signaling 49-53.

The restored lysosomal acidity observed in both *in vitro* and *in vivo* models is particularly significant, as lysosomal alkalinization has been recognized as a central pathological feature in TBI 54, 55. This dysfunction impairs autophagosome-lysosome fusion, promotes accumulation of damaged organelles and toxic proteins, and aggravates oxidative stress 56. The study's observation that AMX restores lysosomal pH via a cAMP-PKA-dependent mechanism, potentially through modulating lysosomal proton pumps and zinc homeostasis, provides an important mechanistic link between cyclic nucleotide signaling and lysosomal-autophagic function 57. Restored lysosomal and autophagic functions is likely mediated through cAMP-PKA signaling, which regulates lysosomal biogenesis and autophagosome-lysosome fusion 58, 59. These findings highlight the multifaceted therapeutic approach of AMX in addressing TBI pathology. Future studies should explore its potential role in activating transcription factor EB (TFEB), a master regulator of lysosomal biogenesis and autophagy 60-63. Neuroinflammation represents another critical target addressed by AMX through suppressing microglial and astrocytic activation 64. The ability of AMX to reduce CD86-positive (M1) microglia and C3-positive reactive astrocytes suggests that it can modulate the glial activation phenotype, shifting it from a pro-inflammatory state to a potentially more neuroprotective state 36, 65, 66. The known involvement of cAMP-PKA signaling in glial regulation, through inhibiting NF-κB and IL-1β transcriptional activity, likely contributes to these effects 67, 68. Importantly, although AMX is a relatively large molecule, previous pharmacokinetic studies and recent evidence suggest that it can cross the BBB under pathological conditions such as neuroinflammation or brain injury, enabling it to exert effects within the central nervous system 29. Furthermore, the observed reduction in the pro-inflammatory cytokines TNF-α and IL-1β in the hippocampus supports this anti-inflammatory mechanism, underscoring the multi-layered regulatory role of cyclic nucleotide signaling in neuroimmune interactions 36, 66, 69. Critically, the improvements in cognitive and motor function following AMX administration, as demonstrated by mNSS and Morris water maze performance, align with these molecular effects 32, 33. Given CREB's central role in transcriptionally regulating neuroplasticity-related genes, this could represent an important mechanism underlying the functional recovery observed in this study 70. By targeting multiple inflammatory pathways, AMX addresses the complex interplay between neuroinflammation and cognitive recovery 28.

In considering the basis of AMX's anti-neuroinflammatory effects, it remains to be determined whether AMX directly suppresses glial activation or if reduced inflammation is a secondary outcome of improved neuronal survival. Several studies indicate that AMX can directly modulate inflammatory signaling in glial cells. For example, Phan Van *et al.* reported that AMX attenuated LPS-induced microglial activation by downregulating NF-κB/STAT3 pathways in BV2 cells and reduced Iba1⁺ microgliosis *in vivo* 29. Similarly, in astrocytes, AMX's inhibition of TBK1 has been shown to diminish pro-inflammatory cytokine production and neuroinflammatory responses after spinal cord injury 71. Consistent with these findings, Yang *et al.* observed that post-seizure AMX treatment markedly decreased activated microglia and astrocytes (Iba1, GFAP) and lowered cytokine levels, supporting the notion of a direct glia-targeted anti-inflammatory action 72. On the other hand, it is also plausible that AMX's neuroprotective effect indirectly contributes to dampened inflammation, as by preserving neuronal viability and limiting cellular damage signals, AMX could secondarily reduce reactive microgliosis and astrogliosis. Given this bidirectional interplay between neurons and glia, the observed attenuation of neuroinflammation with AMX likely reflects both a direct suppression of glial inflammatory activity and an indirect effect whereby improved neuronal health fails to trigger as robust a glial response.

While this study underscores the therapeutic potential of AMX in TBI, several limitations should be acknowledged. The current investigation was limited to specific doses and time points, leaving the long-term efficacy and safety of AMX unaddressed. Future studies are warranted to establish optimal dosing regimens and to assess AMX's effects in more clinically relevant and heterogeneous models of TBI. Moreover, investigating AMX's interaction with additional molecular pathways such as TFEB-mediated lysosomal biogenesis and CREB-driven neurogenesis may provide deeper insights into its multifaceted mechanisms of action. Finally, translational studies are essential for evaluating its pharmacokinetics, safety profile, and therapeutic efficacy in human subjects, particularly given the complexity of and variability in TBI in clinical populations.

While our study demonstrates the neuroprotective effects of AMX in a rodent model of TBI, several limitations should be noted. First, rodent models do not fully replicate the complexity and heterogeneity of human TBI, which may limit clinical translatability. Second, we employed a single dose and time point for AMX administration; future studies should explore dose-response relationships and therapeutic time windows. Third, although we identified several molecular pathways affected by AMX, a more detailed mechanistic investigation, particularly one using genetic or pharmacological pathway-specific tools, is warranted. Fourth, our current analyses focus primarily on histological and biochemical endpoints; additional assessments of long-term functional and cognitive outcomes are needed. Lastly, potential off-target effects of AMX were not addressed in this study, and further evaluation of its safety profile is required. Despite these limitations, our findings provide important preclinical evidence supporting the therapeutic potential of AMX for treating TBI.

Despite these limitations, our findings indicate that AMX holds significant neuroprotective potential for TBI treatment by modulating inflammatory and oxidative stress pathways. It is important to note, however, that AMX is currently FDA-approved only in the topical form as an oral paste for treating aphthous ulcers and has not been approved for systemic use. While its pharmacological properties have prompted investigation into systemic applications for various inflammatory and metabolic disorders, the systemic administration of PDE4 inhibitors, including agents such as AMX, is commonly associated with adverse effects such as nausea, vomiting, and gastrointestinal discomfort. These well-documented side effects raise important considerations for potentially repurposing AMX in systemic or CNS-targeted applications. For TBI, where BBB integrity is transiently disrupted, there is a heightened possibility of central effects, but this also necessitates caution regarding dose selection and tolerability. Therefore, additional pharmacokinetic and safety studies are essential to determine whether AMX can be optimized for systemic administration while minimizing potential side effects. Future studies should investigate its efficacy across varying injury severities, extended time windows, and multiple dosing regimens to optimize its therapeutic application. Additionally, evaluating long-term effects on cognitive and functional recovery will be critical for assessing clinical relevance. Given its existing safety profile in topical indications, AMX presents a strong candidate for drug repurposing in TBI, provided these translational hurdles can be addressed. Future translational research, including studies in large animal models and early-phase clinical trials, will be necessary to fully establish its feasibility and therapeutic potential in human populations.

## Supplementary Material

Supplementary figures and video legends.

Supplementary videos.

## Abbreviations

TBI: Traumatic brain injury
cAMP: cyclic adenosine monophosphate
PKA: Protein kinase A
PDE: Phosphodiesterase
AMX: Amlexanox
BBB: Blood-brain barrier
LAMP2: Lysosome-associated membrane protein 2
MAP2: Microtubule-associated protein 2
PERK: Protein kinase RNA-like endoplasmic reticulum kinase
eIF2α: eukaryotic initiation factor 2 alpha
p38 MAPK: p38 mitogen-activated protein kinases
ROS: Reactive oxygen species
ARRIVE: Animal research: reporting *in vivo* experiments
mNSS: modified neurological severity score
MWM: Morris water maze
PFA: Paraformaldehyde
FJB: Fluoro-Jade B
CA1, CA3: Cornu Ammonis 1, Cornu Ammonis 3
GCL: Granule cell layer
4HNE: 4-hydroxyl-2-nonenal
GFAP: Glial fibrillary acidic protein
Iba-1: Ionized calcium-binding adapter molecule 1
NeuN: Neuronal nuclear protein
DAPI: 4′,6-diamidino-2-phenylindole
EZ-CYTOX: Enhanced cell viability assay kit
SDS-PAGE: Sodium dodecyl sulfate-polyacrylamide gel electrophoresis
TBS-T: Tris-buffered saline with tween
RIPA Buffer: Radio-immunoprecipitation assay buffer
TNF-α: Tumor Necrosis Factor-alpha
IL-1β: Interleukin-1 beta
