# Supplementary material for: Amlexanox Ameliorates Traumatic Brain Injury by Restoring Autophagy-Lysosomal Function via cAMP Signaling Modulation
Source: Int J Biol Sci. 2025 Jul 6;21(10):4467–84. doi: 10.7150/ijbs.111216 (PMC12320245; doi:10.7150/ijbs.111216)
Supplement: Supplementary file 1 — Supplementary figures and video legends. [file ijbsv21p4467s1.pdf]

Supplementary information for International Journal of Biological Sciences.

Amlexanox Ameliorates Traumatic Brain Injury by Restoring Autophagy–Lysosomal Function via cAMP Signaling Modulation

Seo Young Woo[1], Min Kyu Park[1], A Ra Kho[2,3], Hyun Wook Yang[1], Hyun Ho Jung[1], Jaewoo Shin[4], Minwoo Lee[5], Ha Na Kim[6], Jae Young Koh[6], Bo Young Choi[7,8,]* and Sang Won Suh[1,]*

Author information

[1]Department of Physiology, Hallym University College of Medicine, Chuncheon 24252, Republic of Korea; 1wsy@naver.com (S.Y.W.), bagmingyu50@gmail.com (M.K.P.), akqjqtj5@naver.com (H.W.Y.), wjdgusgh1021@gmail.com (H.H.J.)

[2]Neuroregeneration and Stem Cell Programs, Institute for Cell Engineering, [3]Department of Neurology, Johns Hopkins University School of Medicine, Baltimore, MD 21205, USA; akho3@jhu.edu (A.R.K.)

[4]Medical Device Development Center, Daegu-Gyeongbuk Medical Innovation Foundation (K-MEDI Hub), Daegu 41061, Republic of Korea; neuron.shin@gmail.com (J.S.)

[5]Department of Neurology, Hallym Neurological Institute, Hallym University Sacred Heart Hospital, Anyang 14068, Republic of Korea; neuromlee@hallym.or.kr (M.L.)

[6]Neural Injury Lab, Biomedical Research Center, Asan Institute for Life Sciences, Asan Medical Center, Seoul, Korea; kimhn_79@naver.com (H.N.K.), jkko@amc.seoul.kr (J.Y.K.)

[7]Department of Physical Education, [8]Institute of Sport Science, Hallym University, Chuncheon 24252, Republic of Korea

*To whom correspondence should be sent
Bo Young Choi, PhD
Department of Physical Education, Hallym University, College of Natural Sciences, 1 Hallymdaehak-gil, Chuncheon 24252, Republic of Korea
Phone (82) 10-8888-0646 Fax (82) 33-248-2258
e-mail: bychoi@hallym.ac.kr

Sang Won Suh, MD, PhD
Department of Physiology, Hallym University College of Medicine, 1 Hallymdaehak-gil, Chuncheon 24252, Republic of Korea
Phone (82-10) 8573-6364 Fax (82-33) 256-3426
e-mail: swsuh@hallym.ac.kr
RS-2021-NR062028, RS-2025-00520396

Supplemental material

Additional file 1: **Video 1, Video 2, Video 3, and Video 4**.

Data collected from behavioral assessment using the mNSS in rats with TBI demonstrate the potential therapeutic effect of AMX. One of the assessment criteria in the mNSS is the beam balance test, which we videoed in this study. In the sham group, all subjects showed good balance control. However, the rats in the TBI group showed impaired balance compared with those in the sham group. Upon comparing the TBI groups, we observed that the rats in the TBI–AMX group performed better than those in the TBI–vehicle group in terms of balance control. This suggested that AMX improved balance control in TBI rats. Overall, the behavioral assessment data provide conclusive results for using the mNSS in evaluating the potential therapeutic effect of AMX in TBI.

A

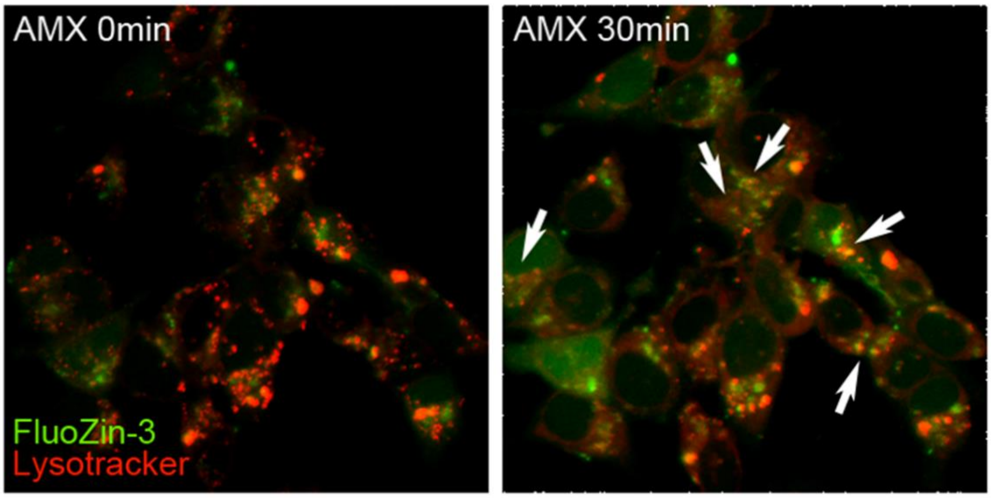

Additional file 2: **Supplementary file 1. Lysosomal zinc levels increased after exposure to AMX.**

(A) Fluorescence photomicrographs of astrocytes stained with FluoZin-3 (green) and Lysotracker DND-99 (red). This demonstrates that exposure to AMX leads to increased zinc levels within lysosomes.

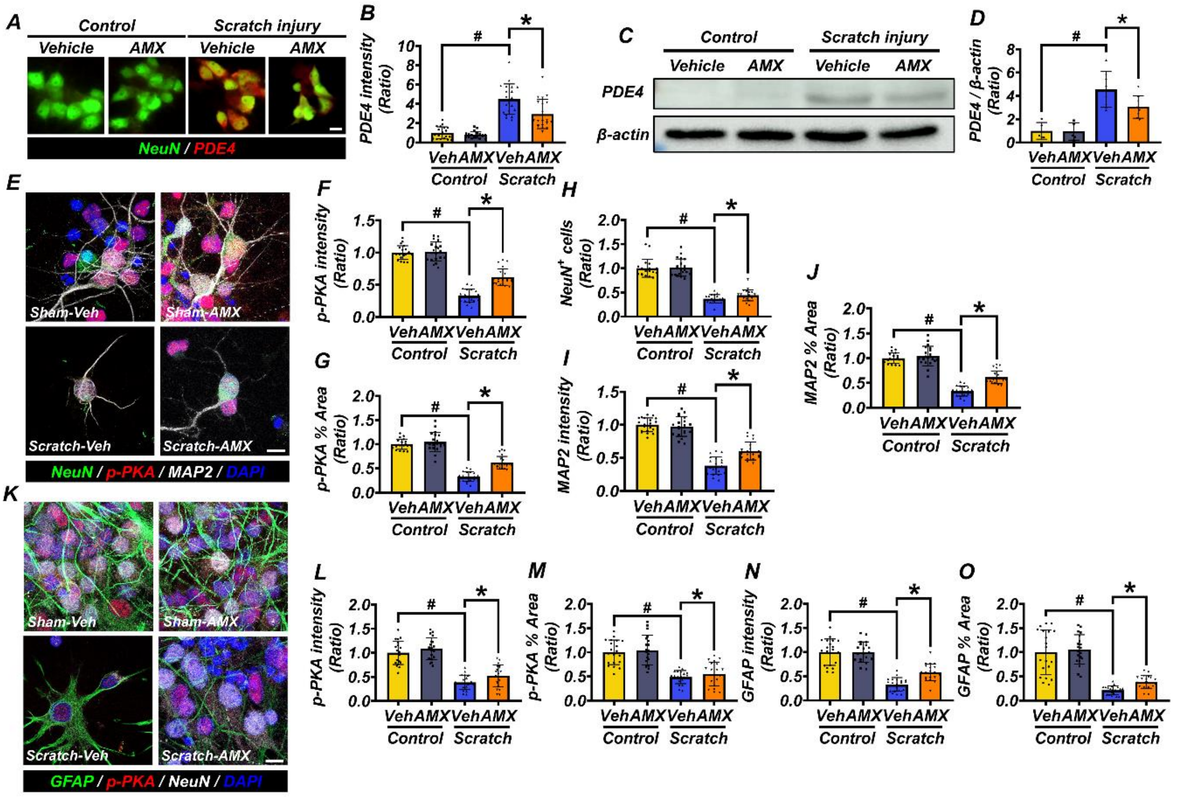

Additional file 3: **Supplementary file 2. AMX reduces PDE4 expression and restores cAMP/PKA signaling after scratch injury in primary neurons and astrocytes.**

(A) Representative immunofluorescence images showing co-staining of NeuN (green, neuronal marker) and PDE4 (red) in primary hippocampal neurons, 24 hours after scratch injury. Scale bar = 20 μm. **(B)** Quantification of PDE4 fluorescence intensity (n = 20/group). # $P < 0.05$ vs. sham; * $P < 0.05$ vs. vehicle-treated TBI (Mean ± SD; Kruskal–Wallis test, Bonferroni post hoc: $\chi^2 = 57.727$, df = 3, $p < 0.001$). **(C)** Representative Western blot images of PDE4 protein levels in primary neurons 24 hours after scratch injury. **(D)** Quantification of Western blot results for PDE4 expression (n = 5 for sham, n = 8 for TBI groups). # $P < 0.05$ vs. sham; * $P < 0.05$ vs. vehicle-treated TBI (Mean ± SD; Kruskal–Wallis test, Bonferroni post hoc: $\chi^2 = 19.312$, df = 3, $p < 0.001$). **(E)** Triple immunofluorescence staining of primary hippocampal neuron and astrocyte co-cultures 24 hours after scratch injury. Cells were stained for NeuN (green), p-PKA (red) and MAP2 (white). Nuclei are stained with DAPI (blue). Scale bar = 50 μm. # $P < 0.05$ vs. sham; * $P < 0.05$ vs. vehicle-treated TBI (Mean ± SD; Kruskal–Wallis test, Bonferroni post hoc: $\chi^2 = 19.312$, df = 3, $p < 0.001$). **(F–J)** Quantitative bar graphs showing p-PKA intensity **(F)**, p-PKA % area **(G)**, NeuN-positive cell count **(H)**, MAP2 intensity **(I)**, and MAP2 % area **(J)** (n = 20/group). # $P < 0.05$ vs. control; * $P < 0.05$ vs. vehicle-treated scratch (Mean ± SD; Kruskal–Wallis test, Bonferroni post hoc: p-PKA intensity: $\chi^2 = 64.330$, df = 3, $p < 0.001$. p-PKA % area: $\chi^2 = 64.643$, df = 3, $p < 0.001$. MAP2 intensity: $\chi^2 = 61.409$, df = 3, $p < 0.001$. MAP2 % area: $\chi^2 = 64.694$,

df = 3, p < 0.001. NeuN-positive cell count: χ^2 = 60.567, df = 3, p < 0.001). **(K)** Triple immunofluorescence staining of primary hippocampal neuron and astrocyte co-cultures 24 hours after scratch injury. Cells were stained for GFAP (green, astrocytes), p-PKA (red), and NeuN (white, neurons). Nuclei were counterstained with DAPI (blue). Scale bar = 50 μm. **(L–O)** Quantitative bar graphs showing p-PKA intensity **(L)**, p-PKA % area **(M)**, GFAP intensity **(N)**, and GFAP % area **(O)**. # P < 0.05 vs. control; * P < 0.05 vs. vehicle-treated scratch (Mean ± SD; Kruskal–Wallis test with Bonferroni correction: p-PKA intensity: χ^2 = 55.604, df = 3, p < 0.001. p-PKA % area: χ^2 = 47.010, df = 3, p < 0.001. GFAP intensity: χ^2 = 56.530, df = 3, p < 0.001. GFAP % area: χ^2 = 57.426, df = 3, p < 0.001).

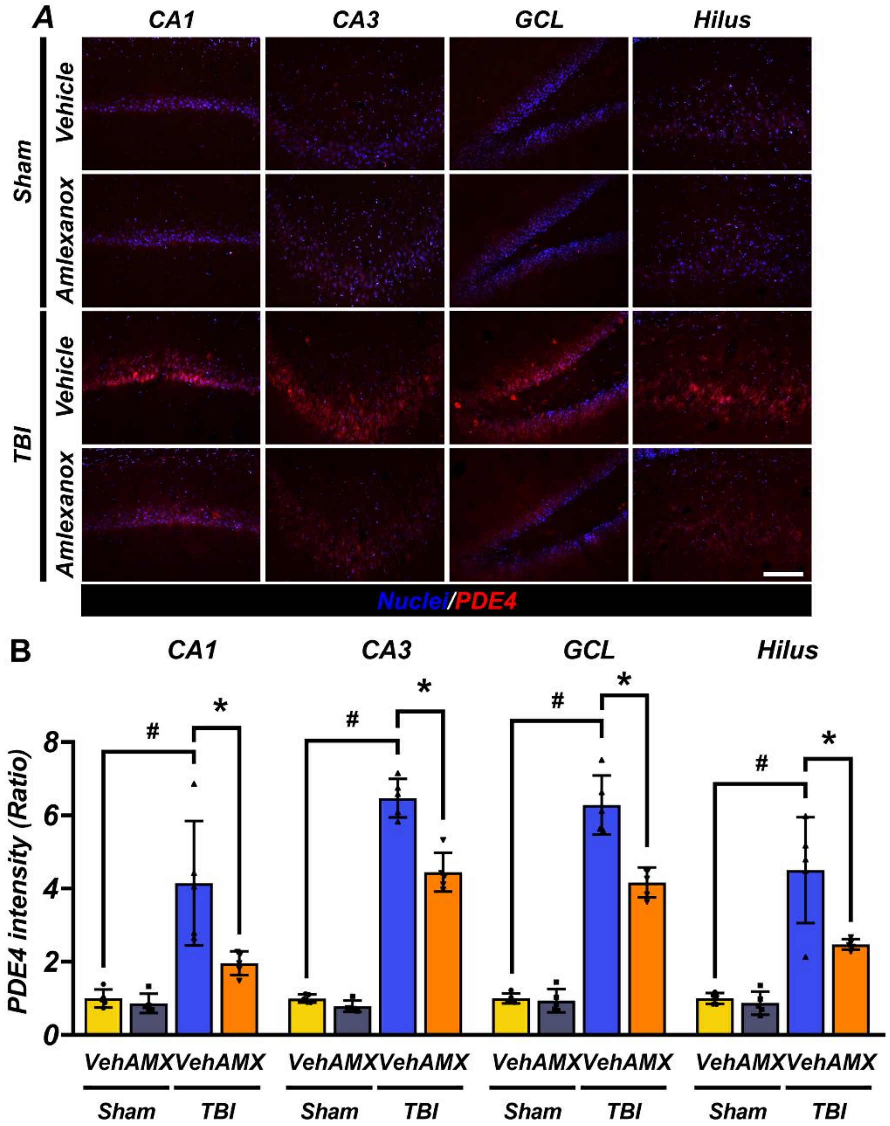

Additional file 4: **Supplementary file 3. AMX reduces PDE4 expression after TBI.**

(A) Immunofluorescence images showing PDE4 (red) staining in the hippocampal region CA1, CA3, GCL, and hilus, 3 hours after TBI. Nuclei are counterstained with DAPI (blue). Scale bar: 100 µm. **(B)** Bar graph showing PDE4 intensity quantification (n=5/sham, n=9-10/TBI) # $P < 0.05$ vs. sham; * $P < 0.05$ vs. vehicle-treated TBI (Mean ± SD; Kruskal–Wallis, Bonferroni: CA1 $\chi^2 = 16.554$, df = 3, p = 0.001; CA3: $\chi^2 = 17.103$, df = 3, p = 0.001; GCL: $\chi^2 = 16.211$, df = 3, p = 0.001; hilus: $\chi^2 = 15.274$, df = 3, p = 0.002).

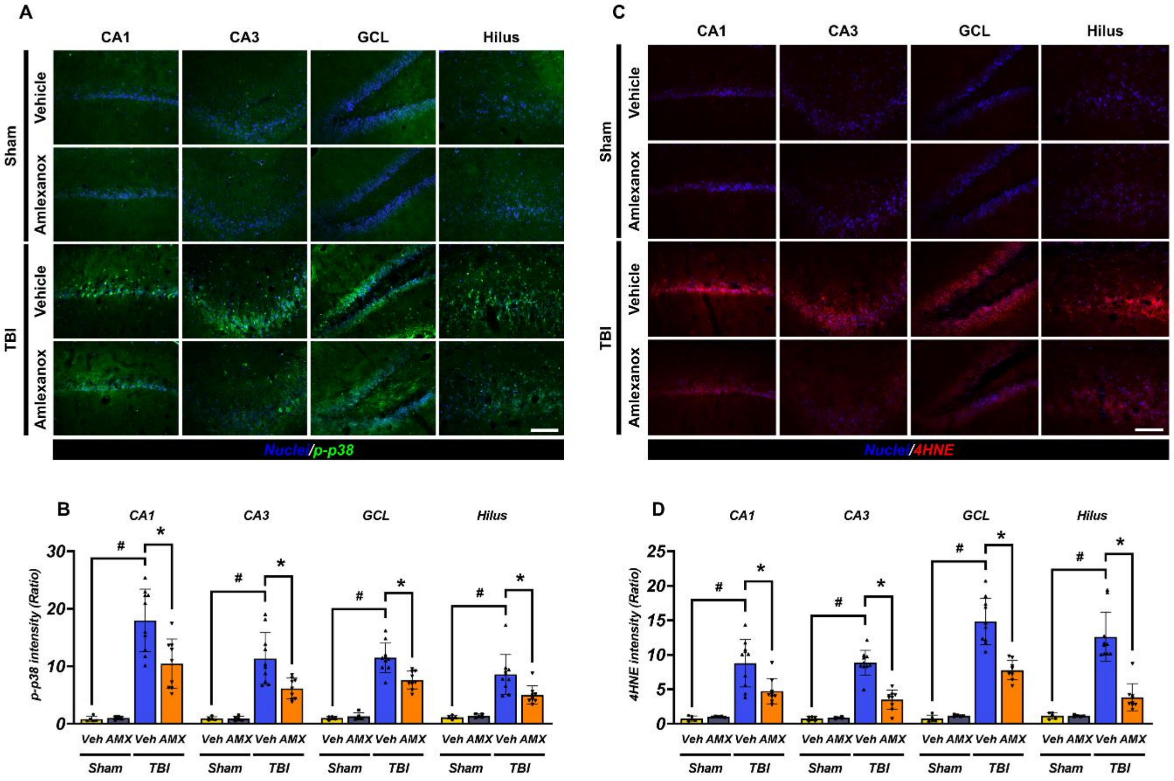

Additional file 5: **Supplementary file 4. AMX reduces cellular stress expression after TBI.**

(A) Immunofluorescence images of the hippocampus (CA1, CA3, GCL, hilus) show p-p38 (green) 24 hours after TBI. Nuclei are counterstained with DAPI (blue). Scale bar: 100 µm. **(B)** Bar graph showing p-p38 intensity quantification (n=5/sham, n=9-10/TBI). # $P < 0.05$ vs. sham; * $P < 0.05$ vs. vehicle-treated TBI (Mean ± SD; Kruskal–Wallis, Bonferroni: CA1: $\chi^2 = 21.854$, df = 3, $p < 0.001$; CA3: $\chi^2 = 22.172$, df = 3, $p < 0.001$; GCL: $\chi^2 = 23.693$, df = 3, $p < 0.001$; Hilus: $\chi^2 = 22.338$, df = 3, $p < 0.001$). **(C)** Immunofluorescence images of the hippocampus (CA1, CA3, GCL, and hilus) show 4HNE staining (red) for lipid peroxidation 24 hours after TBI. Nuclei are counterstained with DAPI (blue). Scale bar: 100 µm. **(D)** Bar graph showing 4HNE intensity quantification (n=5/sham, n=9-10/TBI). # $P < 0.05$ vs. sham; * $P < 0.05$ vs. vehicle-treated TBI (Mean ± SD; Kruskal–Wallis, Bonferroni: CA1: $\chi^2 = 21.910$, df = 3, $p < 0.001$; CA3: $\chi^2 = 22.930$, df = 3, $p < 0.001$; GCL: $\chi^2 = 25.207$, df = 3, $p < 0.001$; hilus: $\chi^2 =$

24.909, df = 3, p < 0.001).

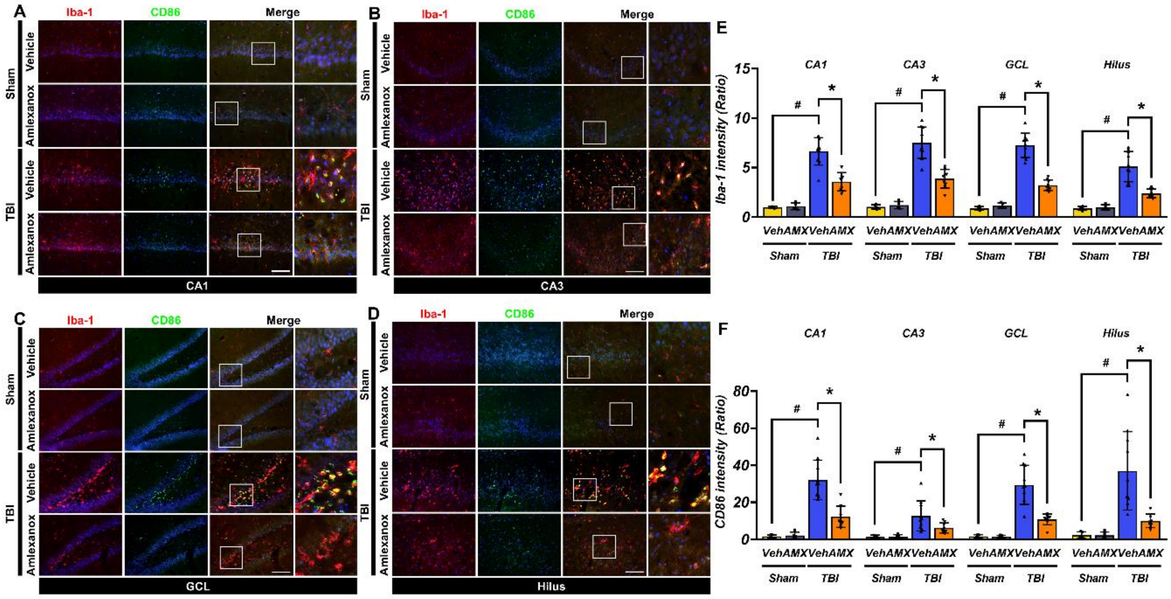

Additional file 6: **Supplementary file 5. AMX reduces microglia activation after TBI.**

(A-D) Images of CA1 **(A)**, CA3 **(B)**, GCL **(C)**, and hilus **(D)** with Iba-1 (red) and CD86 (green) staining for microglia, with merged images included 24 hours after TBI. Nuclei are DAPI-stained (blue). Scale bar: 100 μm. **(E)** Bar graphs quantifying Iba-1 intensity (n=5/sham, n=9-10/TBI). # P < 0.05 vs. sham; * P < 0.05 vs. vehicle-treated TBI (Mean ± SD; Kruskal–Wallis, Bonferroni: CA1: $\chi^2 = 23.671$, df = 3, p < 0.001; CA3: $\chi^2 = 24.496$, df = 3, p < 0.001; GCL: $\chi^2 = 25.295$, df = 3, p < 0.001; hilus: $\chi^2 = 24.937$, df = 3, p < 0.001). **(F)** Bar graphs quantifying CD86 intensity (n=5/sham, n=9-10/TBI). # P < 0.05 vs. sham; * P < 0.05 vs. vehicle-treated TBI (Mean ± SD; Kruskal–Wallis, Bonferroni: CA1: $\chi^2 = 24.149$, df = 3, p < 0.001; CA3: $\chi^2 = 21.284$, df = 3, p < 0.001; GCL: $\chi^2 = 24.385$, df = 3, p < 0.001; hilus: $\chi^2 = 24.385$, df = 3, p < 0.001).

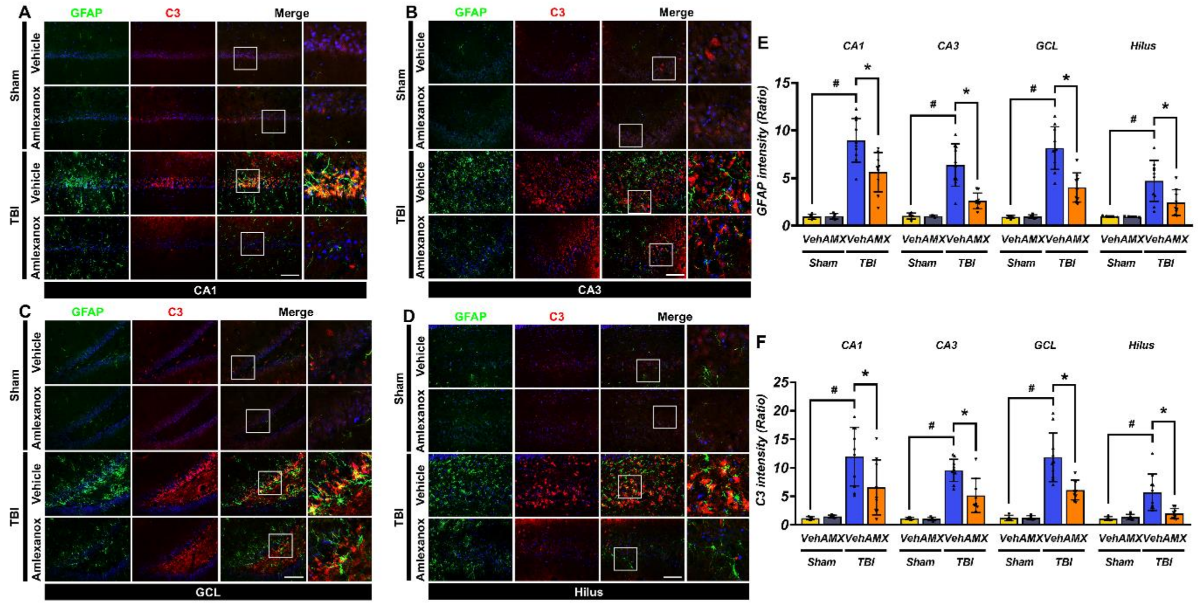

Additional file 7: **Supplementary file 6. AMX reduces astrocyte activation after TBI.**

(A-D) Images of CA1 **(A)**, CA3 **(B)**, GCL **(C)**, and hilus **(D)** with GFAP (green) and C3 (red) staining for astrocytes, 24 hours after TBI. Nuclei are DAPI-stained (blue). Scale bar: 100 µm. **(E)** Bar graphs quantifying GFAP intensity (n=5/sham, n=9-10/TBI). # $P < 0.05$ vs. sham; * $P < 0.05$ vs. vehicle-treated TBI (Mean ± SD; Kruskal–Wallis, Bonferroni: CA1: $\chi^2 = 22.379$, df = 3, $p < 0.001$; CA3: $\chi^2 = 23.272$, df = 3, $p < 0.001$; GCL: $\chi^2 = 23.441$, df = 3, $p < 0.001$; hilus: $\chi^2 = 21.163$, df = 3, $p < 0.001$). **(F)** Bar graphs quantifying C3 intensity (n=5/sham, n=9-10/TBI). # $P < 0.05$ vs. sham; * $P < 0.05$ vs. vehicle-treated TBI (Mean ± SD; Kruskal–Wallis, Bonferroni: CA1: $\chi^2 = 19.545$, df = 3, $p < 0.001$; CA3: $\chi^2 = 22.568$, df = 3, $p < 0.001$; GCL: $\chi^2 = 23.693$, df = 3, $p < 0.001$; Hilus: $\chi^2 = 19.856$, df = 3, $p < 0.001$).

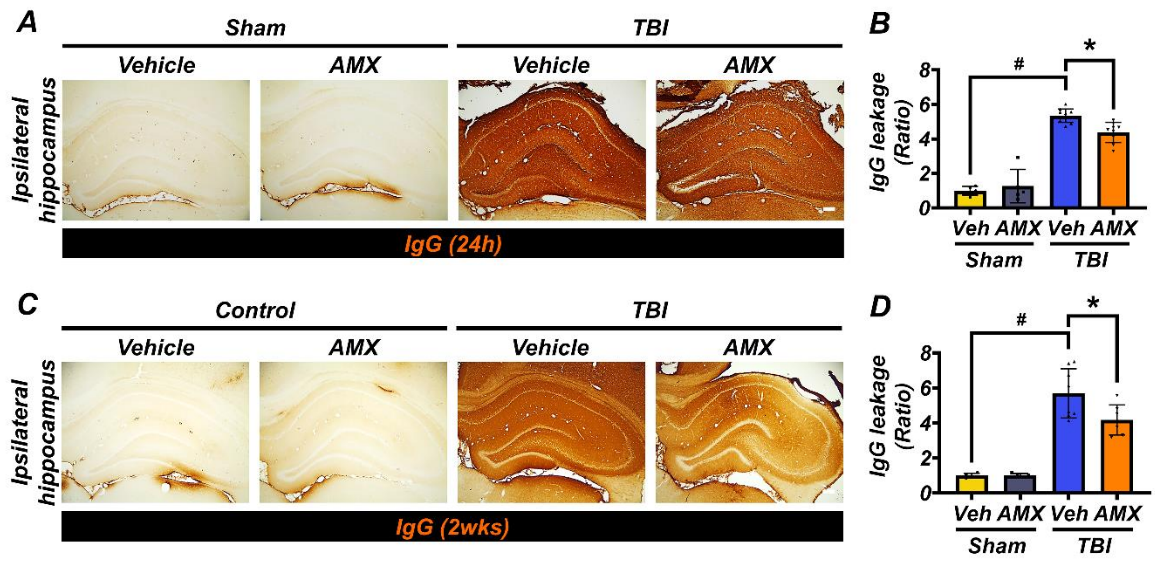

Additional file 8: **Supplementary file 7. AMX attenuates TBI-induced BBB disruption.**

(A) Representative immunofluorescence images showing IgG leakage in the ipsilateral hippocampus as an indicator of BBB disruption at 24 hours post-TBI. Increased IgG extravasation is observed in the vehicle-treated TBI group, while AMX treatment reduces this leakage. Scale bar = 100 μm. **(B)** Quantification of IgG fluorescence intensity in the ipsilateral hippocampus at 24 hours post-TBI. n = 5 (sham group), n = 9–10 (TBI groups). # $P < 0.05$ vs. sham; * $P < 0.05$ vs. vehicle-treated TBI (Mean ± SD; Kruskal–Wallis test, Bonferroni post hoc: $\chi^2 = 23.660$, df = 3, $p < 0.001$). **(C)** Representative immunofluorescence images showing IgG leakage in the ipsilateral hippocampus as an indicator of BBB disruption at 2 weeks post-TBI. **(D)** Quantification of IgG intensity in the ipsilateral hippocampus at 2 weeks post-TBI. n = 5 (sham group), n = 7–8 (TBI groups). # $P < 0.05$ vs. sham; * $P < 0.05$ vs. vehicle-treated TBI (Mean ± SD; Kruskal–Wallis test, Bonferroni post hoc: $\chi^2 = 18.912$, df = 3, $p < 0.001$).

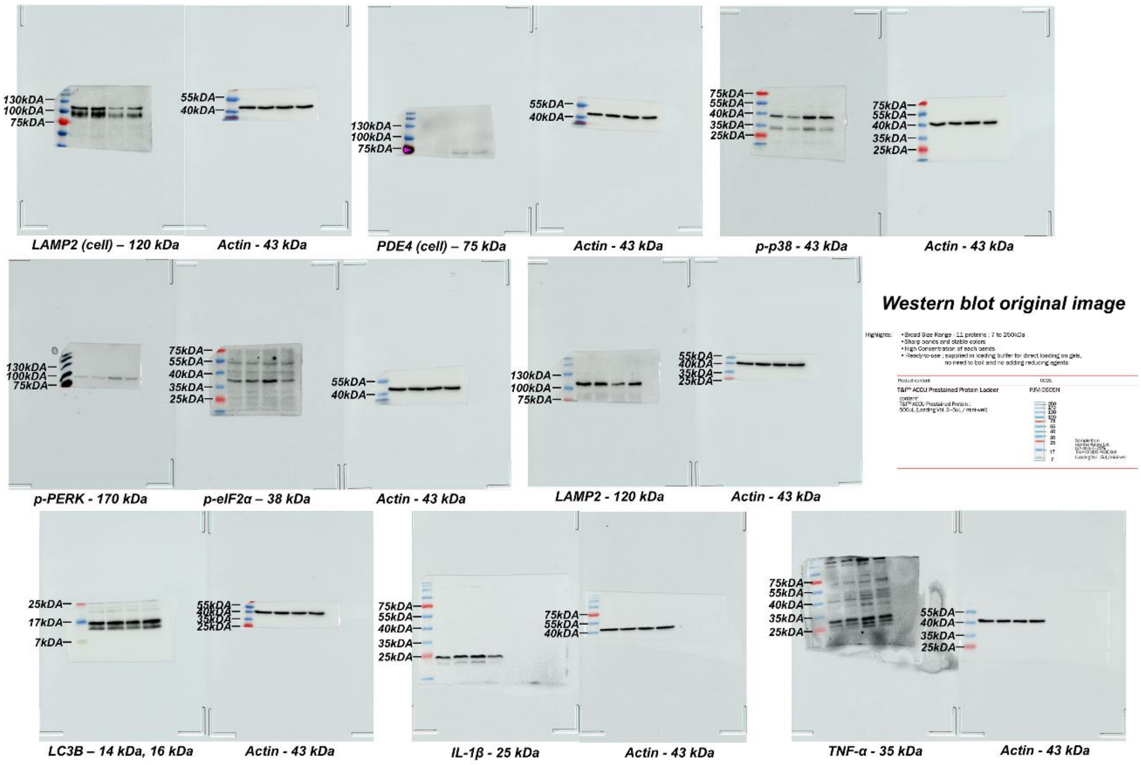

Additional file 9: **Western blot original file.**
